# Global, regional and national burden of anxiety disorders from 1990 to 2019: results from the Global Burden of Disease Study 2019

**DOI:** 10.1017/S2045796021000275

**Published:** 2021-05-06

**Authors:** Xiaorong Yang, Yuan Fang, Hui Chen, Tongchao Zhang, Xiaolin Yin, Jinyu Man, Lejin Yang, Ming Lu

**Affiliations:** 1 Clinical Epidemiology Unit, Qilu Hospital of Shandong University, Jinan, China; 2 Clinical Research Center of Shandong University, Qilu Hospital, Cheeloo College of Medicine, Shandong University, Jinan, China; 3 Department of Epidemiology and Health Statistics, School of Public Health, Cheeloo College of Medicine, Shandong University, Jinan, China; 4 Department of Psychology, Qilu Hospital of Shandong University, Jinan, China

**Keywords:** Anxiety disorders, bullying victimisation, global burden, systematic analysis

## Abstract

**Aims:**

Anxiety disorders are widespread across the world. A systematic understanding of the disease burden, temporal trend and risk factors of anxiety disorders provides the essential foundation for targeted public policies on mental health at the national, regional, and global levels.

**Methods:**

The estimation of anxiety disorders in the Global Burden of Disease Study 2019 using systematic review was conducted to describe incidence, prevalence and disability-adjusted life years (DALYs) in 204 countries and regions from 1990 to 2019. We calculated the estimated annual percentage change (EAPC) to quantify the temporal trends in anxiety disorders burden by sex, region and age over the past 30 years and analysed the impact of epidemiological and demographic changes on anxiety disorders.

**Results:**

Globally, 45.82 [95% uncertainty interval (UI): 37.14, 55.62] million incident cases of anxiety disorders, 301.39 million (95% UI: 252.63, 356.00) prevalent cases and 28.68 (95% UI: 19.86, 39.32) million DALYs were estimated in 2019. Although the overall age-standardised burden rate of anxiety disorders remained stable over the past three decades, the latest absolute number of anxiety disorders increased by 50% from 1990. We observed huge disparities in both age-standardised burden rate and changing trend of anxiety disorders in sex, country and age. In 2019, 7.07% of the global DALYs due to anxiety disorders were attributable to bullying victimisation, mainly among the population aged 5–39 years, and the proportion increased in almost all countries and territories compared with 1990.

**Conclusion:**

Anxiety disorder is still the most common mental illness in the world and has a striking impact on the global burden of disease. Controlling potential risk factors, such as bullying, establishing effective mental health knowledge dissemination and diversifying intervention strategies adapted to specific characteristics will reduce the burden of anxiety disorders.

## Introduction

The anxiety disorders constitute the leading prevalent mental disorders in the world (Stein *et al*., 2017), which are estimated as responsible for about 28.68 million disability-adjusted life years (DALYs) in the Global Burden of Diseases (GBD) 2019 Study, especially for adolescents aged 10–24 years (ranking sixth) (GBD 2019 Diseases and Injuries Collaborators, 2020). People suffering from anxiety disorders usually experience excessive fear, nervousness, avoiding threats that in the environment or perceived by themselves (Craske *et al*., 2009), and are often accompanied by autonomic dysfunction, such as palpitations, dizziness and insomnia (Craske *et al*., 2017). Anxiety disorders are receiving more and more attention, due to the high incidence of anxiety disorders, the early age of onset (Casey and Lee, 2015), the tendency to relapse for a long time and the disabling nature (Bandelow and Michaelis, 2015). More studies have also confirmed that one out of every 14 people meets the diagnostic criteria for anxiety disorder (Craske and Stein, 2016). Considering the high burden and high prevalence associated with anxiety disorders, it is very meaningful and necessary for the formulation of health care policies and plans. Unfortunately, services for mental disorders, such as anxiety are often neglected and are not a global health priority, especially when compared to cancer, cardiovascular diseases. Therefore, the allocation of medical resources for anxiety disorders in many countries is not in proportion to the actual burden (Whiteford *et al*., 2013).

The incidence of anxiety disorders is on the rise worldwide, but the huge geographic disparity represents the complexity in the intervention of anxiety disorders. For example, the lifetime prevalence of anxiety disorders is 0.1% in Nigeria and 8.6% in Canada (Stein *et al*., 2017; Edwards *et al*., 2021). Therefore, it is necessary to grasp the latest spatial distribution and temporal trends of anxiety disorders across the world. Comprehensive and accurate regional and national anxiety disorder data are the basic prerequisites for policy-makers to allocate limited resources and formulate policies rationally. Also, it is worth noting that more and more people are suffering from health loss due to anxiety disorders. Evidence-based psychological and drug therapy is more likely to attract people's attention because it can directly benefit or reduce losses. Although reducing the prevalence through effective treatment would be a desirable approach to avoid the burden, reasonable and effective prevention programs can better reduce these avoidable medical expenses (Baxter *et al*., 2014).

How to estimate the disease burden objectively is based on the accurate epidemiological description of the disease and its sequelae. Previous studies paid more attention to the factors included in the diagnosis of anxiety disorder, such as gender, age, region, disease severity etc. Of course, these factors must be taken into account. Furthermore, reducing morbidity wound be a direct way to averting the burden, such as early detection to reduce the prevalence, especially in children and young people, which may help to reduce the severity and persistence of symptoms (McGorry *et al*., 2011). However, these are closely related to the medical burden caused by the disease itself. Therefore, a more effective approach to burden reduction is based on the methods and conclusions of these previous studies, and also includes the exploration of closely related risk factors. This will expand the perspective for the formulation of relevant policies and bring benefits to more people. Unfortunately, the global spatio-temporal pattern and risk factors of anxiety disorders are still unclear, which has great public health significance for the control of anxiety disorders.

The GBD 2019 Study, a systematical worldwide epidemiological study, quantifies the morbidity, mortality, disability of 369 diseases along with 87 risk factors by location, sex, age and year, which provides a unique opportunity to understand the state of anxiety. In this study, we summarised the incidence, prevalence, DALYs and secular trends of anxiety disorders by gender and age group in 204 countries from 1990 to 2019 based on the GBD 2019 Study. Our findings would help increase the world's attention to anxiety disorders, as well as design targeted strategies for the prevention and intervention of anxiety disorders adapting the specific characteristics in different regions.

## Methods

### Data source

The exhaustive original data sources and fitting methods of the GBD 2019 Study have been delineated in previous studies (GBD 2019 Diseases and Injuries Collaborators, 2020; GBD 2019 Risk Factors Collaborators, 2020), and the analysis process and repeatable statistical codes for estimating anxiety disorders could be retrieved from the supporting website (http://ghdx.healthdata.org/gbd-2019/code/nonfatal-2). Here we presented the methods specific to the estimation of anxiety disorders. Each step used in the current study to analyse the GBD database complies with the Guidelines for Accurate and Transparent Health Estimates Reporting (GATHER) statements (Stevens *et al*., 2016). For the GBD 2019 assessment, anxiety disorders were claimed by the following codes ICD-10: F40-42, F43.0, F43.1, F93.0-93.2, F93.8 and DSM-IV-TR: 300.0-300.3, 208.3, 309.21, 309.81, which covered panic disorder, agoraphobia, specific phobia, social phobia, obsessive–compulsive disorder, posttraumatic stress disorder and generalised anxiety disorder. The prevalence, incidence, remission, duration and/or excess mortality associated with anxiety disorders were searched via the three stages involving electronic searches of the peer-reviewed literature in PsycInfo, Embase and PubMed databases, the grey literature, and expert consultation. The following search terms were listed: ‘panic disorder’, ‘agoraphobia’, ‘social phobia’, ‘generalised anxiety disorder’, ‘obsessive compulsive disorder’, ‘posttraumatic stress disorder’, ‘anxiety disorder’, ‘OCD’, ‘GAD’, ‘PTSD’ and ‘epidemiology’, ‘incidence’, ‘prevalence’, ‘mortality’, ‘remission’ and ‘duration’. According to GBD inclusion criteria, a total of 219 original data sources about anxiety disorders were identified for this assessment of anxiety disorders. The extracted data were further divided by age and sex using the Meta-Regression with Bayesian priors, Regularisation and Trimming (MR-BRT) analysis. The estimation of anxiety disorders with known biases was adjusted or crosswalked accordingly prior to DisMod-MR 2.1. The burden in locations with no available data was fitted by considering the mean war mortality rate in the previous 10 years and Gallup negative experience index using the DisMod-MR meta-regression model. We exacted data on the anxiety disorder burden by sex and 5-year age group in 204 countries and territories from 1990 to 2019 from the Institute for Health Metrics and Evaluation (http://ghdx.healthdata.org/gbd-results-tool). In order to describe the disease burden of anxiety disorders in different geographic units, the 204 countries and territories were categorised into five regions according to their socio-demographic index (SDI, a composite indicator of income per person, years of education and fertility), namely, low, low-middle, middle, high-middle and high SDI regions. In addition, the world was further geographically divided into 21 GBD areas, such as high-income Asia Pacific, Central Latin America and Central Europe, which were also simplified into seven super GBD regions, such as high-income regions. Based on a well-established inclusion criterion for a risk–outcome pair in GBD 2019 Study, only bullying victimisation was judged to have sufficient evidence to prove a causal relationship with anxiety disorders as an outcome among identified 87 behavioral, environmental and occupational, and metabolic risk factors. The disease burden attributable to risk factors was estimated via the comparative risk assessment framework, which includes the estimation of risk–outcome pairs, relative risks, theoretical minimum risk exposure level and population attributable fractions.

### Statistical analysis

The age-standardised incidence rate (ASIR), age-standardised prevalence rate (ASPR) and age-standardised DALYs rate were applied to quantify the difference of anxiety disorders burden by historical period, sex and location, to avoid the difference in age compositions of the populations. The 95% uncertainty intervals (UIs) for every metric in the GBD study were calculated based on the 25th and 975th ordered values of 1000 random draws of the posterior distribution. We further calculated the estimated annual percentage change (EAPC) to describe the temporal trend in various age-standardised rates (ASRs) of anxiety disorders burden (Yang *et al*., 2020). We performed a regression model fitting the natural logarithm of the ASR with the calendar year, namely, ln (ASR) = *α* + β* calendar year + ɛ, to estimate the EAPC with its 95% confidence interval (CI) based on the formula of 100 × (exp (*β*) − 1). We used the Spearman rank correlation to quantify the relationship between the EAPCs in anxiety burden and the baseline burden in 1990 and the SDI in 2019 at the national level. The ASRs of anxiety disorders in 1990 could serve as an exemplification for the baseline disease reservoir, and the SDI in 2019 could reflect the availability and level of health care in each country. All statistical analyses in this study were performed using R program version 4.0.3 (https://www.R-project.org/), and the two-sided *p* value <0.05 was considered statistically significant.

## Results

### Global burden and temporal trend in anxiety disorders

Globally, the estimated newly-diagnosed anxiety disorders patients increased from 31.13 (95% UI: 25.08, 37.92) million in 1990 to 45.82 (95% UI: 37.14, 55.62) million in 2019, with a relatively stable ASIR of about 5.8 (95% UI: 4.68, 7.03) cases per 1000 population over the past 30 years (Table 1). The accumulated anxiety disorder patients worldwide increased from 194.92 (95% UI: 165.10, 231.23) million in 1990 to 301.39 (95% UI: 252.63, 356.00) million in 2019, and kept a stable ASPR with around 37.8 (95% UI: 31.94, 44.77) per 1000 population during the period (online Supplementary Table S1). Likely, in 2019, an estimated 28.68 (95% UI: 19.86, 39.32) million DALYs worldwide attributable to anxiety disorders, compared with the DALYs of 18.66 (95% UI: 12.9, 25.55) million in 1990, with the age-standardised DALYs rate at 3.60 (95% UI: 2.51, 4.92) per 1000 population (online Supplementary Table S2).

**Table 1.** Incidence and age-standardised incidence rate per 1000 people for anxiety disorders in 1990 and 2019, and its estimated annual percentage change from 1990 to 2019

Variables	1990		2019		1990–2019
	Incident cases No. × 10^6^ (95% UI)	ASIR per 1000 No. (95% UI)	Incident cases No. × 10^6^ (95% UI)	ASIR per 1000 No. (95% UI)	EAPC in ASIR No. (95% CI)
Overall	31.13 (25.08, 37.92)	5.79 (4.68, 7.03)	45.82 (37.14, 55.62)	5.85 (4.74, 7.1)	0.02 (0, 0.04)
Sex					
Males	12.92 (10.49, 15.72)	4.81 (3.93, 5.79)	19.38 (15.78, 23.41)	4.9 (4.01, 5.92)	0.06 (0.01, 0.11)
Females	18.2 (14.57, 22.34)	6.81 (5.45, 8.3)	26.44 (21.23, 32.27)	6.84 (5.49, 8.36)	−0.01 (−0.05, 0.03)
SDI region					
High SDI	5.57 (4.48, 6.82)	6.79 (5.47, 8.31)	6.77 (5.45, 8.28)	7.11 (5.7, 8.73)	0.06 (−0.06, 0.18)
High-middle SDI	6.78 (5.49, 8.19)	5.81 (4.71, 6.98)	8.26 (6.68, 9.97)	5.83 (4.75, 7.02)	0.01 (−0.01, 0.03)
Middle SDI	10.19 (8.2, 12.46)	5.84 (4.73, 7.02)	14.3 (11.58, 17.24)	5.85 (4.76, 7.03)	0.11 (0.06, 0.15)
Low-middle SDI	5.87 (4.69, 7.23)	5.38 (4.34, 6.57)	9.8 (7.88, 12.06)	5.44 (4.42, 6.63)	0.14 (0.08, 0.19)
Low SDI	2.7 (2.11, 3.39)	5.42 (4.33, 6.72)	5.91 (4.64, 7.44)	5.31 (4.24, 6.56)	0.12 (0.04, 0.19)
GBD region					
High-income Asia Pacific	0.82 (0.66, 0.99)	4.65 (3.75, 5.59)	0.74 (0.6, 0.89)	4.34 (3.51, 5.29)	−0.26 (−0.35, −0.16)
High-income North America	2.14 (1.73, 2.61)	7.46 (6.04, 9.02)	2.86 (2.31, 3.52)	8.06 (6.44, 9.87)	0.04 (−0.22, 0.3)
Western Europe	2.89 (2.3, 3.59)	7.91 (6.27, 9.75)	3.09 (2.49, 3.78)	7.91 (6.29, 9.77)	0.09 (0.06, 0.12)
Australasia	0.17 (0.13, 0.21)	8.21 (6.41, 10.34)	0.23 (0.18, 0.29)	8.49 (6.61, 10.77)	0.14 (0.07, 0.2)
Tropical Latin America	1.33 (1.07, 1.61)	8.62 (6.95, 10.33)	2.29 (1.83, 2.81)	9.89 (7.91, 12.08)	0.51 (0.27, 0.76)
Andean Latin America	0.3 (0.23, 0.38)	7.8 (6.05, 9.9)	0.51 (0.39, 0.65)	7.88 (6.1, 10.07)	0.05 (0.04, 0.06)
Central Latin America	0.91 (0.72, 1.13)	5.53 (4.42, 6.8)	1.56 (1.24, 1.95)	6.09 (4.84, 7.58)	0.33 (0.21, 0.45)
Southern Latin America	0.36 (0.29, 0.44)	7.17 (5.72, 8.82)	0.48 (0.4, 0.58)	7.3 (6.05, 8.72)	0.06 (0, 0.13)
Caribbean	0.23 (0.18, 0.3)	6.56 (5.12, 8.31)	0.32 (0.25, 0.4)	6.69 (5.2, 8.46)	0.08 (0.07, 0.1)
Eastern Europe	1.16 (0.94, 1.4)	5.07 (4.09, 6.1)	1.05 (0.85, 1.27)	5.04 (4.08, 6.06)	−0.01 (−0.04, 0.02)
Central Europe	0.64 (0.51, 0.79)	5.08 (4.02, 6.3)	0.57 (0.46, 0.7)	5.06 (4.04, 6.24)	−0.01 (−0.02, 0.01)
Central Asia	0.25 (0.2, 0.32)	3.75 (2.94, 4.69)	0.35 (0.27, 0.44)	3.72 (2.91, 4.65)	−0.05 (−0.06, −0.04)
North Africa and Middle East	2.71 (2.15, 3.4)	7.52 (6.03, 9.27)	4.95 (3.87, 6.24)	7.83 (6.16, 9.81)	0.13 (0.1, 0.16)
South Asia	5.09 (4.09, 6.21)	4.92 (3.98, 5.95)	9.22 (7.44, 11.16)	4.98 (4.04, 5.97)	0.22 (0.06, 0.37)
Southeast Asia	2.59 (2.06, 3.2)	5.54 (4.47, 6.8)	4.04 (3.24, 4.94)	5.79 (4.64, 7.1)	0.11 (0.08, 0.14)
East Asia	6.92 (5.61, 8.36)	5.58 (4.57, 6.64)	7.57 (6.27, 9.02)	5.25 (4.34, 6.2)	−0.33 (−0.41, −0.25)
Oceania	0.04 (0.03, 0.05)	6.11 (4.8, 7.59)	0.08 (0.06, 0.11)	6.26 (4.89, 7.86)	0.07 (0.06, 0.09)
Western Sub-Saharan Africa	0.93 (0.73, 1.16)	4.93 (3.97, 6.06)	2.3 (1.81, 2.88)	4.99 (4.02, 6.13)	0.09 (0.04, 0.14)
Eastern Sub-Saharan Africa	1.03 (0.79, 1.29)	5.71 (4.51, 7.12)	2.34 (1.78, 2.96)	5.75 (4.53, 7.2)	0.01 (−0.02, 0.04)
Central Sub-Saharan Africa	0.32 (0.24, 0.42)	6.05 (4.71, 7.65)	0.79 (0.6, 1.02)	6.04 (4.66, 7.69)	0.01 (0, 0.01)
Southern Sub-Saharan Africa	0.3 (0.24, 0.37)	5.77 (4.65, 6.97)	0.46 (0.37, 0.57)	5.76 (4.62, 7.01)	0.01 (−0.02, 0.03)

No., number; ASIR, age-standardised incidence rate; UI, uncertainty interval; EAPC, estimated annual percentage change; and CI, confidential interval.

### Variation in anxiety disorders burden at regional and national level

The new anxiety disorder cases were greatest in middle SDI regions [14.30 (95% UI: 11.58, 17.24)] million in 2019, but the leading ASIR was observed in the high SDI region (7.11/1000) (Table 1). For the GBD regions, the ASIR was greater than 8.0/1000 in Tropical Latin America, Australasia, high-income North America and Western Europe, all with a slightly increasing trend. On the contrary, the lowest ASIR was found in Central Asia with 3.72/1000, followed by high-income Asia Pacific (4.34/1000).

**Fig. 1.**
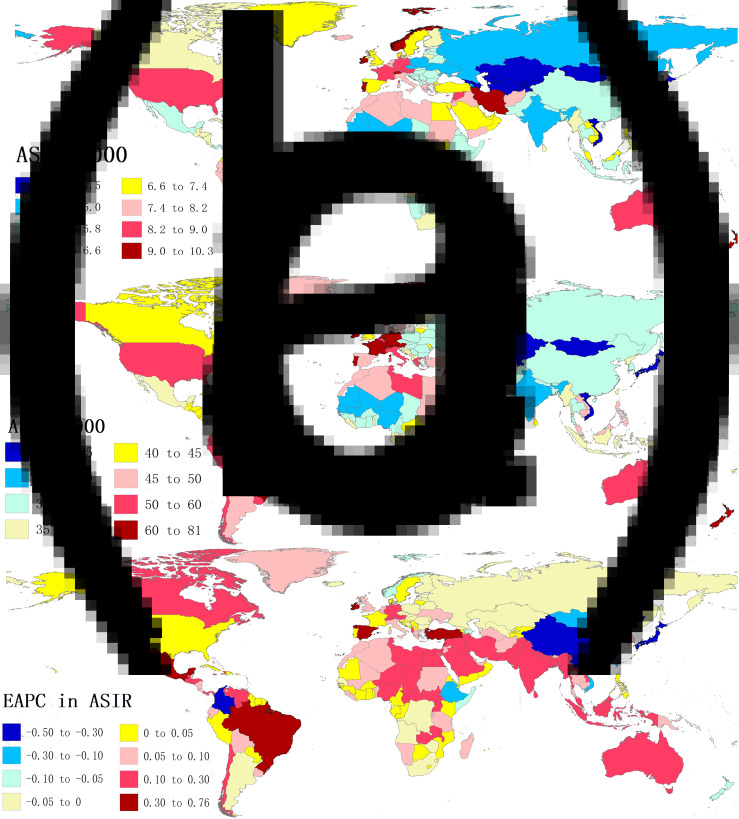
The global disease burden of anxiety disorders in 204 countries and territories: (a) the ASIR in 2019, (b) the ASPR in 2019 and (c) the EAPC in ASIR of anxiety disorders from 1990 to 2019. ASIR, age-standardised incidence rate; ASPR, age-standardised prevalence rate and EAPC, estimated annual percentage change.

The variety of ASIR of anxiety disorders was close to three times across the world in 2019, with the highest ASIR observed in Iran (10.27/1000), and the lowest rate observed in Uzbekistan (3.49/1000). Besides, the ASIR in 2019 exceeding 8/1000 was observed in other 21 countries, including Portugal, Brazil, New Zealand, Norway etc. (Fig. 1a, online Supplementary Table S3). Conversely, the ASIR was lower than 5/1000 in Kyrgyzstan, Kazakhstan, Mongolia and other 21 countries (Fig. 1, online Supplementary Table S4). The geographic distribution of ASPR and age-standardised DALYs rate of anxiety disorders in 2019 were highly consistent with the distribution of ASIR (online Supplementary Tables S3 and S4, Fig. 1, online Supplementary Fig. S1).

The ASIR of anxiety disorders significantly increased in middle, low-middle and low SDI regions from 1990 to 2019 [EAPC = 0.11 (95% CI: 0.06, 0.15), EAPC = 0.14 (95% CI: 0.08, 0.19) and EAPC = 0.12 (95% CI: 0.04, 0.19), respectively], conversely the ASIR remained stable in high and high-middle SDI regions (Table 1). The largest increase in ASIR was observed in Tropical Latin America [EAPC = 0.51 (95% CI: 0.27, 0.76)), followed by Central Latin America and South Asia (EAPC = 0.33 (95% CI: 0.21, 0.45) and EAPC = 0.22 (95% CI: 0.06, 0.37), respectively], whereas East Asia, high-income Asia Pacific and Central Asia presented a downward trend in the ASIRs [lowest EAPC = −0.33 (95% CI: −0.41, −0.25) in East Asia]. The similar trend in ASPR and age-standardised DALYs rate of anxiety disorders were found during the period (online Supplementary Tables S1 and S2).

From 1990 to 2019, the largest annualised growth of ASIR in Mexico [EAPC = 0.76 (95% CI: 0.56, 0.96)], and Brazil had the largest annualised growth rate of ASPR and age-standardised DALYs rate (both EAPCs close to 1.00). Moreover, the EAPC of ASIR exceeding 0.15 was observed in other 19 countries and territories, such as Ireland, Spain, Turkey, Lebanon, Nepal etc. (online Supplementary Table S5, Fig. 1c). Conversely, we found the fastest descent rate of all ASRs was observed in Japan [EAPC in ASIR = −0.50 (95% CI: −0.59, −0.41)], and the EAPC of ASIR less than −0.03 was found in other 20 countries and territories, including China, Colombia, Mongolia, Ethiopia etc. (online Supplementary Table S4, Fig. 1c). Overall, the increased EAPCs in ASPR and age-standardised DALYs rate were greater than those in ASIR (online Supplementary Table S6, Fig. S1).

### Variation in anxiety disorders burden in two sexes and 5-year age groups

**Fig. 2.**
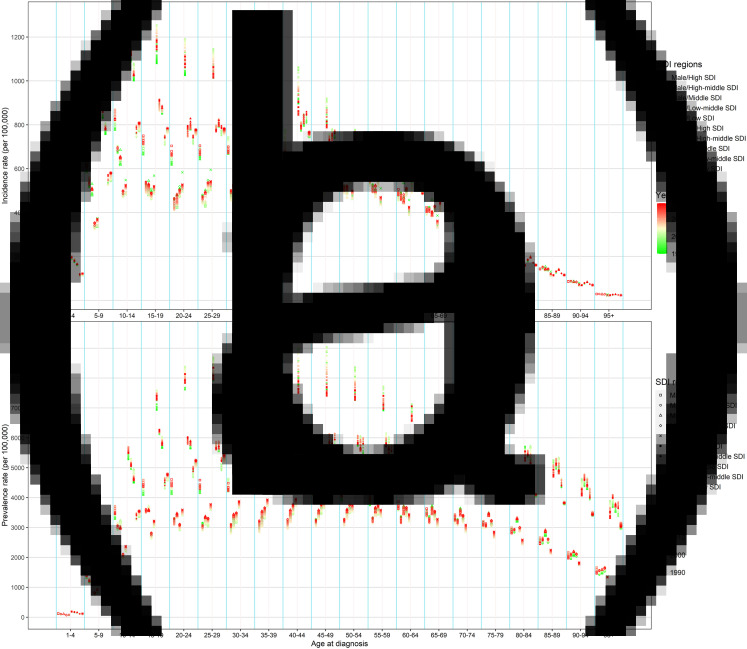
The annual burden of anxiety disorders by different age groups, two sexes and SDI regions, from 1990 to 2019: (a) incidence rate and (b) prevalence rate. SDI, socio-demographic index.

The ASIR of anxiety disorders among women was somewhat greater than men [6.84 (95% UI: 5.49, 8.36) *v*. 4.90 (95% UI: 4.01, 5.92) per 1000 population in 2019] (Table 1), especially among the people aged 5–54 years (online Supplementary Figs S2 and S3, Fig. 2). However, both the ASPR and age-standardised DALYs rate among women were 1.6 times than men across almost all age groups (online Supplementary Figs S2 and S3, Fig. 2). Of note, the gender disparity in disease burden of anxiety disorders presented a slight narrowing trend (Table 1, online Supplementary Tables S1 and S2).

The incidence rate of anxiety disorders rose to a peak at age 10–14 years, remained relatively high level until age 40–44 years, and dropped substantially afterward among both sexes in 2019 (online Supplementary Fig. S2, Fig. 2). Regarding the prevalence rate and DALYs rate, the peak of age susceptibility shifted to 20–50 years, and the prevalence rate and DALYs rate over 55 years old were significantly higher than that of incidence rate (online Supplementary Figs S2 and S3, Fig. 2).

**Fig. 3.**
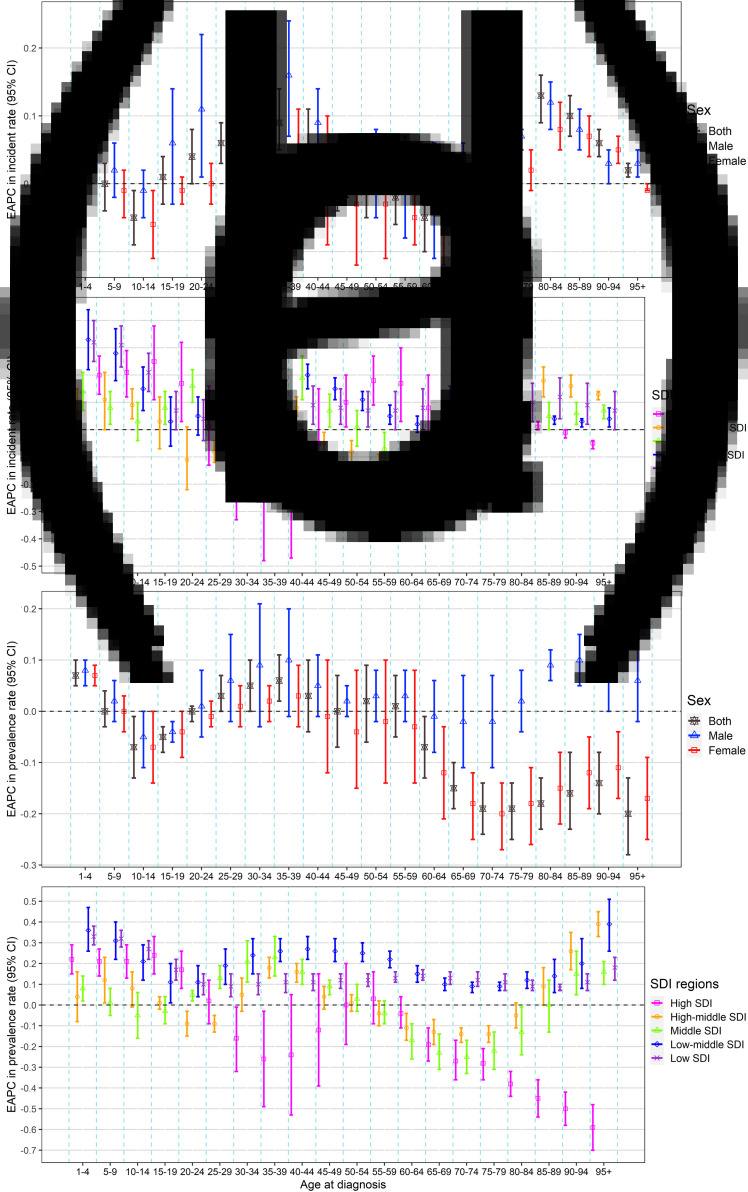
The change of the burden of anxiety disorders by different age groups, sexes and SDI regions, from 1990 to 2019: (a) EAPC in incidence rate by sexes, (b) EAPC in incidence rate by SDI regions, (c) EAPC in prevalence rate by sexes and (d) EAPC in prevalence rate by SDI regions. EAPC, estimated annual percentage change and SDI, socio-demographic index.

From 1990 to 2019, the incidence rate slightly increased among the population aged 20–39 and over 75 years, especially among men and lower SDI regions (Figs 2 and 3). However, the prevalence rate (consistent with DALYs) gradually decreased among the population aged over 60 years, especially among women and high SDI region (Figs 2 and 3, online Supplementary Figs S3 and S4). For the incidence rate, the difference in five SDI regions and sexes focused on the population aged under 50 years (Fig. 2). The prevalence rate of anxiety disorders, similar to DALYs rate, in females was steeper than those in males, especially in the high SDI region (Fig. 2, online Supplementary Fig. S3).

### The correlation between SDI and anxiety disorders burden

**Fig. 4.**
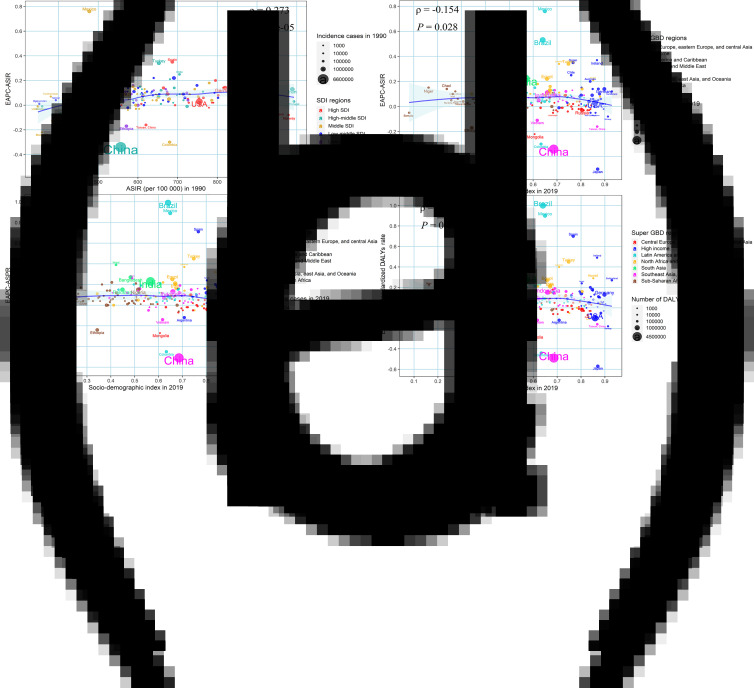
The factors affected the EAPCs in age-standardised burden rate of anxiety disorders from 1990 to 2019, both sexes, at the national level: (a) ASIR in 1990 and EAPC in ASIR, (b) SDI in 2019 and EAPC in ASIR, (c) SDI in 2019 and EAPC in ASPR and (d) SDI in 2019 and EAPC in age-standardised DALYs rate. The circles represent countries and the size of the circle is increased with the number of burdens. The *ρ* indices and *p* values presented were derived from Spearman rank analysis. ASPR, age-standardised prevalence rate; EAPC, estimated annual percentage change; SDI, socio-demographic index; DALYs, disability-adjusted life years and ASIR, age-standardised incidence rate.

We found that the EAPC of ASIR was positively correlated with baseline ASIR in 1990 (*ρ* = 0.273, *p* = 7.9 × 10^−5^) at the national level (Fig. 4a). Further, we analysed the relationship between SDI in 2019 and EAPC values in ASIR, ASPR, and age-standardised DALYs rate in 204 countries or territories, which presented a negative correlation [*ρ* of ASIR: −0.154 (*p* = 0.028), of ASPR: −0.162 (*p* = 0.021), of age-standardised DALYs rate: −0.221 (*p* = 0.001)] (Figs 4b–d). Online Supplementary Fig. S5 shows the relationship between ASIR and SDI over time in 21 GBD regions, expressed in the annual time series of 1990 and 2019. Over time, the ASRs in most GBD regions remained relatively stable, except Tropical Latin America, Southern Latin America, high-income Asia Pacific and East Asia presented a climbing trend beforehand and the following decline afterwards in recent years.

### The anxiety disorders burden attributable to bullying victimisation

**Fig. 5.**
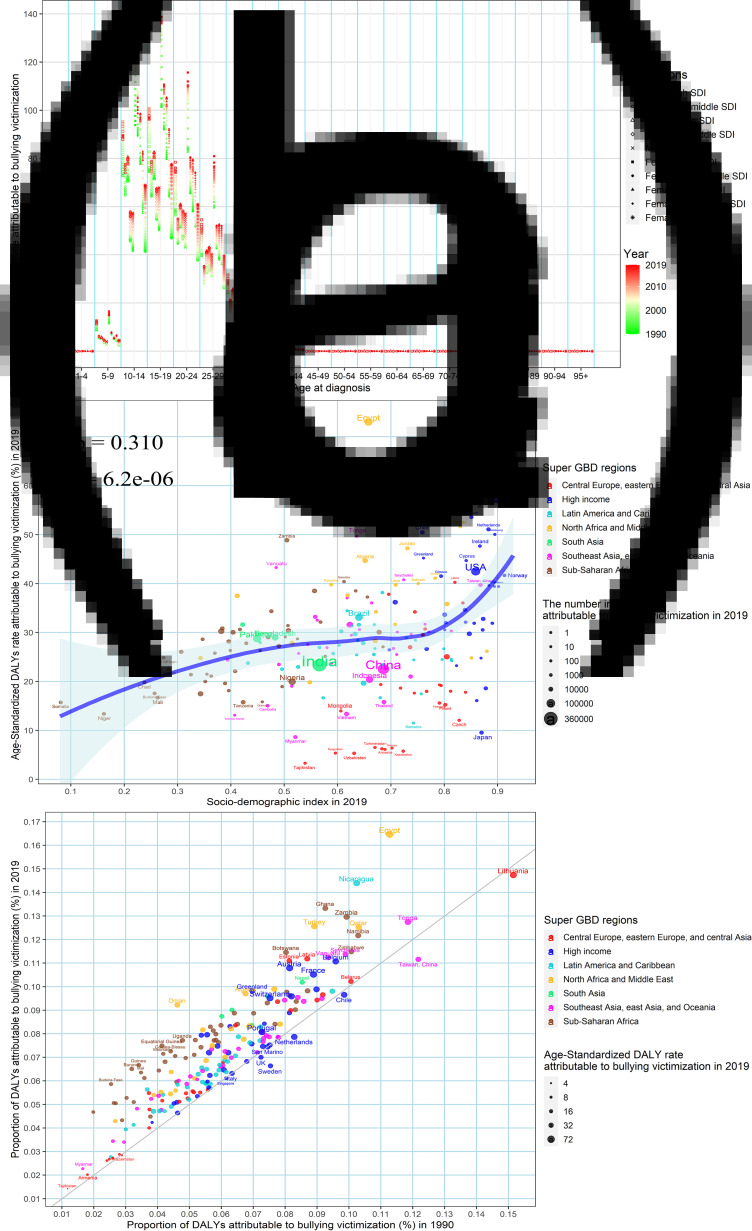
The DALYs of anxiety disorders attributable to bullying victimisation: (a) the annual DALYs rate of anxiety disorders by different age groups, two sexes and SDI regions, from 1990 to 2019, (b) the association between age-standardised DALYs rates of anxiety disorders attributable to bullying victimisation in 2019 and SDI in 2019, (c) the proportion of DALYs of anxiety disorders attributable to bullying victimisation between 1990 and 2019. DALYs, disability-adjusted life years and SDI, socio-demographic index.

Of the global DALYs caused by anxiety disorders, it is estimated that 2.03 (95% UI: 0.59, 4.42) million or 7.07% (95% UI: 2.15%, 14.38%) were attributable to bullying victimisation in 2019, especially among the population aged 5–39 years (Fig. 5a). The age-standardised DALYs rate of anxiety disorders attributable to bullying victimisation in 2019 was positively associated with SDI in 2019 at the national levels (*ρ* = 0.310, *p* = 6.2 × 10^−6^) (Fig. 5b). Besides, the proportion of DALYs due to bullying victimisation increased in almost all countries and territories between 1990 and 2019, except for nine regions, including Taiwan, Sweden, Lithuania etc. (Fig. 5c). The largest proportion attributable to bullying victimisation in 2019 was found in Egypt (0.16), and the proportion exceeding 0.10 was observed in other 20 countries and territories, such as Lithuania, Nicaragua, Ghana, Zambia etc. (Fig. 5c).

## Discussion

Studies demonstrated that anxiety disorders have become a major clinical and public health problem, which were associated with a significantly increased mortality risk. Therefore, the high prevalence of anxiety disorders and the associated excessive mortality have a huge impact on public health (Meier *et al*., 2016). To our best knowledge, we first reported comprehensively the global, regional, and national burden in incidence, prevalence and DALYs of anxiety disorders by sex and age groups along with the temporal trend over the past 30 years in 204 countries and territories. From a global perspective, the number in incidence, prevalence and DALYs of anxiety disorders had increased year by year, but the ASRs remained relatively stable in most countries and territories. However, the current burden and change trend of anxiety disorders vary greatly by region, sex and age group. In particular, in most high-burden areas, the annualised increasing trends were relatively pronounced. Therefore, the systematic understanding of the exact change pattern of anxiety disorders burden is essential for policy-makers to allocate rationally limited medical resources and make adapted prevention and treatment strategies.

Sex differentials were observed in the distribution of anxiety disorders (McLean *et al*., 2011). Our results indicated that the burden of anxiety disorders in women was higher than that of men, with 1.6 times in prevalence and DALYs. Bandelow *et al.* reported that women suffer from anxiety disorders almost twice that of men, and about one-third of women may suffer from anxiety disorders once in their lifetime (Bandelow and Michaelis, 2015). The reason for the increased risk of anxiety in women remains unclear. Generally speaking, innate factors determine that women are more likely to have anxiety than men (Grenier *et al*., 2019), including more sensitivity, insecure and likely to report more traumatic experiences in women than men, especially when facing adverse life events or stress (Vasiliadis *et al*., 2020). Second, there are common features across the world: violence, sexual abuse, antenatal and postnatal stress, the social and cultural environment, and gender inequality(Heise *et al*., 2019; Dworkin, 2020) may put a heavier burden on women. Furthermore, the average income of professional women is much lower than their male counterparts, which also makes women face more pressure and prone to more anxiety (Čermaková *et al*., 2020). In the high SDI region, the prevalence of women is significantly higher than that of men, which may be due to the higher recognition and diagnosis rate of anxiety in high SDI countries. Moreover, it has been suggested that the sex differentials in anxiety disorder were related to genetic and hormonal factors (Li and Graham, 2017; India State-Level Disease Burden Initiative Mental Disorders Collaborators, 2020). Interestingly, there is a slight downward trend in women's anxiety burden in recent years, especially in the high SDI region, which may be related to the fact that women's anxiety is attracting more and more attention.

From 1990 to 2019, we found that the incidence of most age groups gradually increased. This result supports the results of previous studies (Vasiliadis *et al*., 2013). Although there is no data on the prevalence of anxiety across generations, some evidence suggests that the endorsement of anxiety symptoms is increasing (Twenge *et al*., 2010), which may be attributed to improved methods of detection. Studies have shown that the onset of many anxiety disorders is early and predicts later psychopathology (Baxter *et al*., 2014), starting in childhood and adolescence (Craske and Stein, 2016), reaching a peak in middle age, and then tending to decline with age (Grenier *et al*., 2019). So identification of people at risk and interventions at young ages is important treatment considerations (Craske and Stein, 2016). We observed that the incidence rate reaches a peak between 10 and 14 years old, which may be related to childhood abuse (Vachon *et al*., 2015), corporal punishment (Clauss and Blackford, 2012), low socioeconomic status (Moreno-Peral *et al*., 2014), an overprotective or overly harsh parenting style, increased self-consciousness and increased opposition to parents(Beesdo-Baum and Knappe, 2012). Furthermore, the peak of ASPR and DALYs is about 50 years old, and the disease burden of patients over 55 years old is significantly higher than that of ASIR patients. This situation could be attributed to the comorbidity of anxiety disorder and other somatic diseases, lack of early cognition and effective intervention, long-term existence and lifetime recurrence of anxiety disorder. Therefore, mental health education for specific groups (e.g. school-based mental health programmes for students) may be cost effective (Holmes *et al*., 2018), and multi-channel interventions and treatments (e.g. film-based education, internet-based and telephone-based helplines and mental health mobile apps) look forward to development and improvement (Kang *et al*., 2020; White *et al*., 2020; Goodwin *et al*., 2021).

Researches have demonstrated that estimates of the prevalence of anxiety disorders vary from country to country, with the 12-month prevalence ranging from 2.4% in Italy to 29.8% in Mexico. Moreover, the prevalence in the United States and European countries tend to be higher than in other parts of the world (Sourander *et al*., 2011). The present results also showed that the changing trends of anxiety burden vary greatly from country to country and region. In 2019, New Zealand's ASIR reached the highest level, a highly developed country, which may relate to high-level economic conditions, social pressures, cultural environment and an aging population, and high self-awareness of anxiety symptoms. It is worth mentioning that the ASPR of anxiety disorders significantly increased in the lower SDI regions, but decreased slightly among elder patients in the high SDI region, which may partly be attributed to early recognition, accessible and effective treatments.

At present, the aetiology of anxiety disorders is still controversial. Some factors such as parents’ history of mental illness, low socioeconomic status and overprotective parenting styles are considered to be associated with an increased risk of anxiety disorders (Beesdo-Baum and Knappe, 2012; Moreno-Peral *et al*., 2014). Genetic epidemiological studies have found that anxiety disorders have moderate familial aggregation, with heritability is estimated to be 30–50% (Shimada-Sugimoto *et al*., 2015). More evidence has demonstrated that bullying victimisation was strong associated with anxiety (Jadambaa *et al*., 2019). It is worth noting that school bullying incidents have become increasingly prominent, which has become a common problem affecting the physical and mental health of childhood and adolescence (Juvonen and Graham, 2014). Juvonen *et al*. report that about 20–25% of teenagers are directly involved in bullying incidents, and their roles are either assailants, victims or both (Juvonen and Graham, 2014). Our results demonstrated that 7.07% of global DALYs caused by anxiety disorders can be attributed to bullying victimisation in 2019, especially among the population aged 5–39 years. Moreover, the proportion of DALYs due to bullying victimisation increased in most countries and territories between 1990 and 2019. Victims of bullying not only show physical problems such as headaches, stomach pains (Wolke and Lereya, 2014), but also often have mental problems. For example, some patients exhibit suicidal tendencies during adolescence, while others commit theft, drug abuse, drug sales and even anti-social behaviour (Wolke and Lereya, 2015). Therefore, preventing and reducing bullying will become an extremely important and effective measure that the government and society should take into consideration.

If left untreated, anxiety disorders tend to develop chronically, with a waxing and waning pattern of recurrence throughout life, which will inevitably cause a lot of waste of medical resources and social–economic burden (Kessler *et al*., 2010). Early prevention is expected to be very cost effective by offsetting the functional impairments associated with anxiety disorders (Baxter *et al*., 2014). For example, studies have confirmed that computer-assisted and internet-based treatments (e-interventions) are an effective treatment for youth anxiety (Christensen *et al*., 2014; James *et al*., 2020). The current treatment guidelines for anxiety disorders use a combination of psychology and medication (DeMartini *et al*., 2019), and the combination of the two is more effective than a single treatment (Craske and Stein, 2016). Moreover, early identification and elimination of anxiety triggers, such as caffeine, nicotine, stress etc. can help reduce anxiety symptoms (Locke *et al*., 2015). Promote a healthy lifestyle, strengthen physical exercise (Rawson *et al*., 2015), scientifically healthy diet, attach importance to school bullying incidents (Chou *et al*., 2020) and pay attention to the mental health of teenagers and children.

This study comprehensively explained the global burden of anxiety, but it still has some shortcomings due to the restrictions of the GBD 2019 database. First, the GBD estimation on anxiety disorders is reconstructed by mathematical models based on plenty of sources with different quality, which may deviate from the actual data to a certain extent, especially in some underdeveloped regions with extremely scarce prior information, such as Africa and South Asia (GBD 2019 Diseases and Injuries Collaborators, 2020). Second, due to the higher probability of missed diagnosis rate of anxiety in developing countries, there is an unavoidable deviation in the estimation of anxiety burden (Ruscio *et al*., 2017). Moreover, due to the lack of relevant data, we did not estimate the burden of various subtypes of anxiety disorders, such as panic disorder and social phobia. Finally, we only analysed the anxiety burden attributed to bullying victimisation and did not involve an analysis of other potential risk factors of anxiety, such as unmarried, unemployed and low education (Ruscio *et al*., 2017). Future research should focus on this aspect, which will help guide different countries and regions to formulate specific prevention and treatment policies for anxiety.

In conclusion, the overall burden of anxiety disorders is very staggering and continues to increase, and it presents a huge heterogeneity in different sexes, locations and age groups. Understanding the specific characteristics of anxiety disorders burden across the world and reducing risk factors such as bullying, establishing effective mental health knowledge dissemination, improving early diagnosis and performing diversified intervention strategies are of utmost importance to formulate more effective and targeted intervention and control of anxiety disorders.

## Supplementary material

For supplementary material accompanying this paper visit http://dx.doi.org/ 10.1017/S2045796021000275. click here to view supplementary material

## Data

All data could be extracted from the online database (http://ghdx.healthdata.org/gbd-results-tool).
