# Supplementary material for: Global, regional and national burden of anxiety disorders from 1990 to 2019: results from the Global Burden of Disease Study 2019
Source: Epidemiol Psychiatr Sci. 2021 May 6;30:e36. doi: 10.1017/S2045796021000275 (PMC8157816; doi:10.1017/S2045796021000275)
Supplement: Supplementary file 1 [file epssup.zip › S2045796021000275sup001.pdf]

Anxiety Disorders

Flowchart

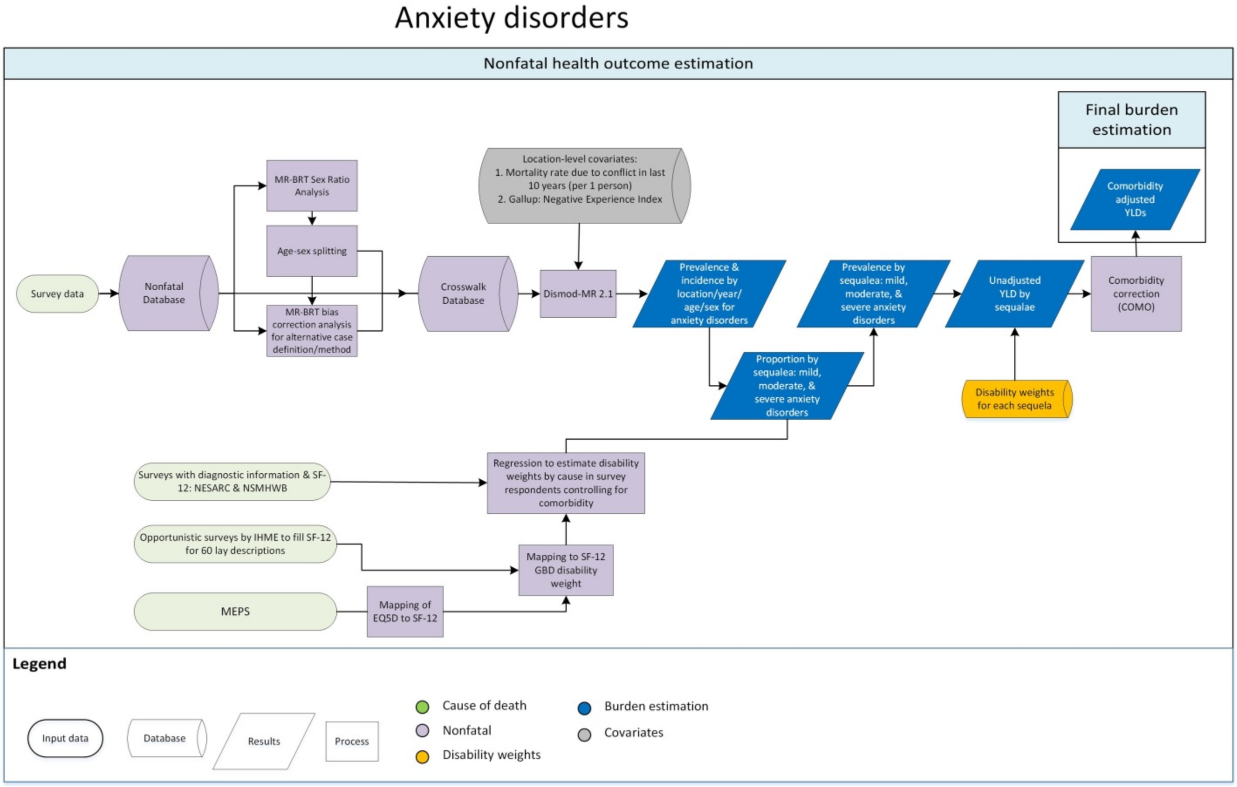

Input Data and Methodological Summary for Anxiety disorders

Case definition

Anxiety disorders are characterised by experiences of intense fear and distress, typically in combination with other physiological symptoms. We aimed to capture all cases of anxiety disorders reaching diagnostic threshold defined by the Diagnostic and Statistical Manual of Mental Disorders (DSM) or the World Health Organization (WHO) International Classification of Diseases (ICD)[1, 2]. Specific anxiety disorders included were: panic disorder, agoraphobia, specific phobia, social phobia, obsessive-compulsive disorder (OCD), posttraumatic stress disorder (PTSD), generalized anxiety disorder (GAD) including overanxious disorder in childhood, separation anxiety disorder (SAD), and anxiety disorder 'not otherwise specified' (NOS). These were identified by the following codes: DSM-IV-TR: 300.0-300.3, 208.3, 309.21, 309.81; ICD-10: F40-42, F43.0, F43.1, F93.0-93.2, F93.8. Excluded were anxiety disorders due to a general medical condition and substance-induced anxiety disorder.

Anxiety disorders were modelled as a single cause for "any" anxiety disorder to avoid the double-counting of individuals meeting criteria for more than one anxiety disorder. Epidemiological estimates reporting an outcome for "any" or "total" anxiety disorders were included in analyses, if they reported on at least three anxiety disorders. This has been further explained in previous publications[3, 4]

Input data

For mental disorders, we update our GBD electronic database searches on a two-year rolling basis. In GBD 2019 a systematic literature review update was conducted to update new epidemiological studies on anxiety disorders published between September 2016 and December 2018. We included studies reporting the prevalence, incidence, remission, duration, and/or excess mortality associated with anxiety disorders. The search was conducted in three stages involving electronic searches of the peer-

reviewed literature (i.e., using PsycInfo, Embase, and PubMed), the grey literature, and expert consultation. The following search terms were used to develop search strings across all databases searched: *'panic disorder', 'agoraphobia', 'social phobia', 'generalised anxiety disorder', 'obsessive compulsive disorder', 'post-traumatic stress disorder', 'anxiety disorder', 'OCD', 'GAD', 'PTSD' and 'epidemiology', 'incidence', 'prevalence', 'mortality', 'remission', 'duration'.*

The search generated 6325 records (after duplicates were removed) across the three electronic databases. The title/abstract screening reduced the number of relevant records to 208 studies, of which 32 studies met criteria for inclusion. An additional 9 studies were identified and extracted through a grey literature search and consultations with experts. Overall, in GBD 2019 we added 41 new studies into the anxiety dataset.

The GBD inclusion criteria stipulated that: (1) the publication year must be from 1980 onward; (2) "caseness" must be based on clinical threshold as established by the DSM or ICD; (3) sufficient information must be provided on study method and sample characteristics to assess the quality of the study; (4) a minimum of 3 (or 2 if occurring during childhood) anxiety disorder subtypes must be included within the overall estimate; and (5) study sample must be representative of the general population (i.e., inpatient or pharmacological treatment samples, case studies, veterans, or refugee samples were excluded). No limitation was set on the language of publications. Methods used in previous systematic reviews have been reported in greater detail elsewhere [3, 4]. Table 1 below summarizes data inputs by parameter for anxiety disorders.

Table 1: Data Inputs for Anxiety disorders morbidity modelling by parameter.

Measure	Total sources	Countries with data
All measures	219	59
Prevalence	199	58
Incidence	1	1
Remission	3	3
Standardized mortality ratio	1	1
Proportion	15	1

Age and sex splitting

The extracted data underwent three types of age and sex splitting processes:

1. Where possible, estimates were further split by sex and age based on the data that was available. For instance, if studies reported prevalence for broad age groups by sex (e.g., prevalence in 15 to 65 year old males and females separately), and also by specific age groups but for both sexes combined (e.g., prevalence in 15- to 30-year-olds, then in 31- to 65-year-olds, for males and females combined); age-specific estimates were split by sex using the reported sex ratio and bounds of uncertainty.
2. A Meta-Regression with Bayesian priors, Regularization, and Trimming (MR-BRT) analysis was used to split the remaining both-sex estimates in the dataset. For each parameter, sex specific estimates were matched by location, age, year and a MR-BRT network meta-analysis was used to estimate pooled sex ratios and bounds of uncertainty. These were then used to split the both sex estimates in the dataset. The male: female prevalence ratio estimated was 0.55 (95% uncertainty interval [UI]: 0.38 – 0.72).
3. Studies reporting prevalence estimates across age groups spanning 25 years or more were split into five-year age groups using the prevalence age pattern estimated by DisMod-MR 2.1.

The DisMod-MR model used to estimate the age pattern did not contain any previously age split data.

Bias corrections / Crosswalks

Estimates with known biases were adjusted / crosswalked accordingly prior to DisMod-MR 2.1. For each crosswalk of interest, pairs of the reference and the alternative estimates were matched by age, sex, location and year. This was done for both within (where possible) and between study pairs. These pairs were then used as inputs in a MR-BRT network meta-analysis. The MR-BRT analysis produced a pooled ratio between the reference estimates and alternative estimates, which was used to adjust all alternative estimates in the dataset. For anxiety disorders, a past year recall ratio was used to adjust all past year recall estimates towards the level they would have been if the estimate had capture point/past-month prevalence. The latter prevalence period is less affected by recall bias. See Table 2 for adjustment factors used for anxiety disorders.

Table 2: MR-BRT Crosswalk Adjustment Factors for Anxiety disorders.

Data input	Reference or alternative case definition	Beta Coefficient, Log (95% UI)	Adjustment factor* (95% UI)	Gamma
Population Survey	Reference: past month or point prevalence			0.23
Population Survey	Alternative: past year prevalence	0.46 (0.01 – 0.91)	1.58 (0.99 – 2.41)	

**Adjustment factor is the transformed Beta coefficient in normal space and can be interpreted as the factor by which the alternative case definition is adjusted to reflect what it would have been if measured as the reference.*

UIs incorporate Gamma which represents the between study variance across all input data in the model. This added uncertainty widens the UIs for crosswalks with significant fixed effects.

Severity splits and disability weights

The GBD disability weight survey assessments are lay descriptions of sequelae highlighting major functional consequences and symptoms. The lay descriptions and disability weights for anxiety disorder severity levels are shown in Table 3. To determine the proportion of people with anxiety disorders within each of the severity levels we used data from The United States' Medical Expenditure Panel Survey (MEPS, conducted in annual waves since 1996)[5], the US National Epidemiological Survey on Alcohol and Related Conditions (NESARC, conducted in two waves from 2001–2002 and 2004–2005)[6], and the Australian National Survey of Mental Health and Wellbeing of Adults (NSMHWB, conducted in 1997)[7]. The proportion of anxiety disorder cases falling within each level of severity was: asymptomatic 28.8% (27.5% – 30.1%), mild 39.3% (34.2% – 44.2%), moderate 19.1% (15.8% – 22.7%) and severe 12.7% (9.2% – 16.7%).

Table 3. Severity distribution for Anxiety disorders in GBD 2019 and the associated disability weight (DW) with that severity.

Severity level	Lay description	DW (95% UI)
Mild	Feels mildly anxious and worried, which makes it slightly difficult to concentrate, remember things, and sleep. The person tires easily but is able to perform daily activities.	0.03 (0.018 – 0.046)
Moderate	Feels anxious and worried, which makes it difficult to concentrate, remember things, and sleep. The person tires easily and finds it difficult to perform daily activities.	0.133 (0.091 – 0.186)
Severe	Constantly feels very anxious and worried, which makes it difficult to concentrate, remember things, and sleep. The person has lost pleasure in life and thinks about suicide.	0.523 (0.362 – 0.677)

Modeling strategy

After the above data processes were applied, DisMod MR 2.1 was used to model the epidemiological data for anxiety disorders. The DisMod-MR modeling strategy for anxiety disorders followed the standard GBD 2019 decomposition structure. At each decomposition step, we compared the new model against the GBD 2017 best model and the best model from the previous step. All substantial changes between models were explored and explained. Adjustments to model priors or the dataset were made where appropriate. Where outliers were identified in the data, we re-assessed the study's methodology and quality before a decision was made to exclude or include the data.

Data across all epidemiological parameters were initially included in the modelling process. The incidence studies reported estimates which were very low relative to the prevalence data. As prevalence studies contributed much greater world coverage than incidence studies, we excluded the incidence data, relying instead on data from the other parameters. We assumed no incidence and prevalence before age 2 and after age 95. This minimum age of onset was corroborated with expert feedback and existing literature on anxiety disorders. Remission was set to a maximum of 0.2, consistent with the data points available.

The following location-level covariates were used to inform the estimation of prevalence in locations with no available data:

1. The mean war mortality rate in the previous 10 years. This covariate identified, for each GBD location, the mean mortality rate due to war and terrorism. It was used given existing evidence that shows a positive association between conflict status and the prevalence for anxiety disorders[8, 9].
2. The Gallup negative experience index. The Gallup initiative conducts comprehensive and comparable national surveys across a wide range of countries worldwide[10]. This index measured respondents' past day experiences of physical pain, worry, sadness, stress and anger. The Gallup covariate was included as a means to test for a correlation between negative emotions at a location level and anxiety disorder prevalence. Data from the Gallup negative experience index was modelled using the Spatio-temporal Gaussian process regression (STGPR) to produce estimates for all years and locations required by DisMod-MR. The log of the modelled output was used as the covariate in DisMod-MR due to skewedness of the data. The relationship detected was as expected, where the higher the negative emotion, the higher the prevalence rate detected.

A summary of covariates and exponentiated values for anxiety disorders are shown in Table 4.

Table 4. Summary of covariates used in the Anxiety disorders DisMod-MR meta-regression model

Covariate	Type	Parameter	Exponentiated beta (95% UI)
Mean war mortality rate in the previous 10 years	Location-level	Prevalence	1.65 (1.07 — 2.54)
Gallup: Negative experience index	Location-level	Prevalence	2.48 (1.80 — 3.61)

Changes between GBD 2017 and GBD 2019

There were five main changes in the GBD 2019 modelling strategy compared to GBD 2017:

1. In GBD 2019 we updated the age splitting by regional pattern methodology by increasing the age threshold for splitting to 25 years (in GBD 2017 it was 20 years). This meant that there were fewer estimates eligible for age splitting in this way. This impacted on the prevalence for some locations which now had fewer age-split estimates informing prevalence estimation.
2. In GBD 2017 bias corrections and sex ratios were estimated by DisMod-MR as part of the prevalence modelling. In GBD 2019 we conducted a MR-BRT analysis to accommodate for study heterogeneity and estimated pooled ratios with 95% UIs as previously discussed. Ratios estimated between 2017 and 2019 were largely consistent, although the UIs derived by MR-BRT tended to be larger. MR-BRT UIs incorporate Gamma which represents the between study variance across all input data in the model. This added uncertainty widens the UIs for crosswalks with significant fixed effects. For example:
    a. The male: female ratio was 0.54 (0.52 – 0.56) in GBD 2017 compared to 0.55 (0.38 – 0.72) in GBD 2019.
    b. The adjustment ratio for past year estimates was 1.48 (1.41 – 1.56) in GBD 2017 compared to 1.58 (0.99 – 2.41) in GBD 2019, leading to a slight overall decrease in adjusted prevalence.
3. The GBD 2017 model included an adjustment ratio (as a study level covariate within DisMod-MR) for estimates derived from school surveys. This covariate/adjustment was excluded in GBD 2019. The school survey adjustment was used in GBD 2017 based on the premise that school surveys might not be representative of the general population, especially in less developed parts of the world. Estimates derived from school surveys were adjusted downwards by 1.54 (1.36 – 1.75) towards the level of estimates from general household surveys. Part of the new GBD 2019 MR-BRT approach was to assess the availability of data for a given study-level covariate to produce robust matched pairs. We were only able to produce a small number of matched pairs for this covariate, primarily from high income countries which would not be representative of other locations. After further review of the literature and discussion with a number of experts in the area, it became apparent that there was insufficient evidence to fully support the direction and magnitude of the GBD 2017 covariate. It also appeared that bias between school surveys and household samples (and the extent to which the latter would be the gold standard) would vary considerably by location. Until more data becomes available to clarify the above, we have excluded this adjustment from the dataset, accepting both types of surveys. The removal of this adjustment from GBD 2019 meant that prevalence derived from student surveys were no longer being adjusted downwards to the extent they were in GBD 2017.
4. In GBD 2019 we included a second location level covariate, Gallup: negative experience index, to further improve the predictive power of the model. The Gallup covariate was significant at 2.48 (1.80 – 3.61). Resulting changes in prevalence by location were in the expected direction.
5. In GBD 2019 we included new epidemiological data from 18 locations (Argentina, Australia, Austria, Denmark, Finland, France, Germany, Lithuania, Mexico, Netherlands, Ningxia,

Portugal, Brazil, Spain, Switzerland, Taiwan, Iran, and United States) which further informed the DisMod-MR model. Some of these studies were from locations where we had no data previously (e.g., Argentina, Portugal, Iran)

While we continue to improve on the data and methods used to estimate the burden of mental disorders, some challenges need to be acknowledged. Firstly, our case definition for anxiety disorder will need to be revised to better capture changes to latest DSM/ICD criteria. Epidemiological estimates reporting an outcome for "any" or "total" anxiety disorders were included in GBD 2019, if they reported on at least three anxiety disorders. Future iterations of GBD will revisit the unique contribution of specific anxiety disorders. Secondly, we still have a large number of locations with no high-quality raw data available. Thirdly, it is difficult to quantify and remove all variation due to measurement error in our epidemiological estimates. Whilst we have improved the methodology used to account for known sources of bias, in some case, we still have very few data points to inform these adjustments. Fourthly, there is a paucity of research on the risk factors of mental disorders which can be used as predictive covariates in our epidemiological models.

References

1. American Psychiatric Association, *Diagnostic and Statistical Manual of Mental Disorders (DSM-IV-TR)*. Fourth Edition, Text Revision ed. 2000, Washington DC: American Psychiatric Association.
2. World Health Organization, *The ICD-10 Classification of Mental and Behavioural Disorders. Clinical descriptions and diagnostic guidelines.* 1992, World Health Organization: Geneva.
3. Baxter, A.J., et al., *Global prevalence of anxiety disorders: a systematic review and meta-regression.* Psychological Medicine, 2013. **43**(05): p. 897-910.
4. Baxter, A.J., et al., *The global burden of anxiety disorders in 2010.* Psychological Medicine, 2014. **44**(11): p. 2363-2374.
5. Health, U.D.o. and H. Services, *Agency for Healthcare Research and Quality, United States. Preventive services task force."Screening and treatment for major depressive disorder in children and adolescents: recommendation statement; March 2009*. 2010.
6. Introduction to the National Epidemiologic Survey on Alcohol and Related Conditions [http://pubs.niaaa.nih.gov/publications/arh29-2/74-78.htm]. Access date 1 December 2014.
7. Australian Bureau of Statistics, *National Survey of Mental Health and Wellbeing of Adults 1997. Canberra: Australian Bureau of Statistics*.
8. Karam, E. and G.M. Bou, *Psychosocial consequences of war among civilian populations.* Current Opinion in Psychiatry 2013. **16**(413–419).
9. Steel, Z., et al., *Association of torture and other potentially traumatic events with mental health outcomes among populations exposed to mass conflict and displacement: a systematic review and meta-analysis.* JAMA, 2009. **302**(5): p. 537-49.
10. Gallup, G., *The Gallup Poll: Public Opinion 2003*. 2004: Rowman & Littlefield.
