# Supplementary material for: Global, regional and national burden of anxiety disorders from 1990 to 2019: results from the Global Burden of Disease Study 2019
Source: Epidemiol Psychiatr Sci. 2021 May 6;30:e36. doi: 10.1017/S2045796021000275 (PMC8157816; doi:10.1017/S2045796021000275)
Supplement: Supplementary file 1 [file epssup.zip › S2045796021000275sup002.docx]

**Global, regional, and national burden of anxiety disorders from 1990 to 2019: Results from the Global Burden of Disease Study 2019**

**Short title: Global analysis of anxiety disorders**

**Xiaorong Yang, PhD^1,2,#^, Yuan Fang, MSc^3,#^, Hui Chen, MPH^1,2^, Tongchao Zhang, PhD^3^, Xiaolin Yin, PhD^3^, Jinyu Man, MSc^3^, Lejin Yang, PhD^4,*^, Ming Lu, PhD, MD^1,2,3,*^**

**Supplementary Materials**

| **Table S1.** Prevalence and age-standardized prevalence rate per 1000 people for anxiety disorders in 1990 and 2019, and its estimated annual percentage change from 1990 to 2019. | | | | | | |
| --- | --- | --- | --- | --- | --- | --- |
| **Variables** | **1990** | | **2019** | | **1990-2019** |  |
|  | **Prevalence cases**  **No.×10^6^ (95% UI)** | **ASPR per 1000**  **No. (95% UI)** | **Prevalence cases**  **No.×10^6^ (95% UI)** | **ASPR per 1000**  **No. (95% UI)** | **EAPC in ASIR**  **No. (95% CI)** | |
| **Overall** | 194.92 (165.1, 231.23) | 37.92 (31.94, 44.77) | 301.39 (252.63, 356) | 37.8 (31.81, 44.73) | -0.02 (-0.05, 0.01) | |
| **Sex** |  |  |  |  |  | |
| Males | 73.43 (61.28, 86.99) | 28.39 (23.89, 33.33) | 113.85 (95.4, 135.15) | 28.6 (23.97, 33.8) | 0.02 (-0.02, 0.07) | |
| Females | 121.49 (101.99, 144.69) | 47.32 (39.83, 56.05) | 187.54 (157.71, 221.6) | 46.95 (39.46, 55.77) | -0.04 (-0.1, 0.02) | |
| **SDI region** |  |  |  |  |  | |
| High SDI | 40.42 (33.99, 48.07) | 46.37 (38.84, 55.3) | 52.41 (44.15, 62.47) | 48.07 (40.17, 57.82) | -0.02 (-0.15, 0.11) | |
| High-middle SDI | 44.36 (37.67, 52.26) | 37.9 (32.22, 44.38) | 58.09 (49.16, 68.18) | 37.54 (31.89, 44.07) | 0.01 (-0.01, 0.03) | |
| Middle SDI | 61.39 (51.48, 73.24) | 37.26 (31.58, 43.74) | 95.17 (80.38, 111.54) | 37.94 (32.21, 44.39) | 0.04 (0, 0.07) | |
| Low-middle SDI | 33.62 (28.07, 40.15) | 33.85 (28.35, 40.22) | 60.59 (50.68, 72.41) | 34.7 (29.09, 40.97) | 0.2 (0.15, 0.25) | |
| Low SDI | 15.01 (12.19, 18.3) | 34.02 (28.02, 41.2) | 34.93 (28.26, 42.72) | 34.95 (28.8, 42.41) | 0.13 (0.09, 0.18) | |
| **GBD region** |  |  |  |  |  | |
| High-income Asia Pacific | 5.19 (4.35, 6.12) | 28.11 (23.55, 33.34) | 5.16 (4.36, 6.06) | 26.16 (21.84, 31.08) | -0.26 (-0.34, -0.19) | |
| High-income North America | 15.55 (13.34, 18.01) | 51.62 (44.35, 60.18) | 21.95 (18.88, 25.64) | 55.6 (46.94, 65.83) | -0.09 (-0.4, 0.23) | |
| Western Europe | 22.57 (18.67, 27.23) | 55.43 (45.76, 67.5) | 26.06 (21.71, 31.45) | 56.27 (46.33, 68.14) | 0.14 (0.08, 0.2) | |
| Australasia | 1.27 (1.01, 1.59) | 59.66 (47.63, 74.95) | 1.8 (1.47, 2.23) | 60.32 (48.85, 74.48) | 0.13 (0.04, 0.21) | |
| Tropical Latin America | 8.39 (7.22, 9.82) | 59 (51.13, 68.36) | 17.76 (15.15, 20.8) | 73.79 (62.96, 86.06) | 0.97 (0.53, 1.41) | |
| Andean Latin America | 1.85 (1.47, 2.35) | 54.03 (43.47, 67.68) | 3.51 (2.84, 4.41) | 54.97 (44.68, 68.93) | 0.08 (0.06, 0.09) | |
| Central Latin America | 5.29 (4.29, 6.46) | 35.21 (29.31, 42.53) | 10.1 (8.34, 12.31) | 39.31 (32.53, 47.83) | 0.36 (0.2, 0.52) | |
| Southern Latin America | 2.53 (2.11, 3.04) | 51.51 (43, 61.89) | 3.57 (3.12, 4.1) | 51.26 (44.6, 58.85) | -0.04 (-0.07, -0.01) | |
| Caribbean | 1.46 (1.15, 1.83) | 42.79 (34.25, 53.55) | 2.15 (1.72, 2.69) | 44.01 (35.22, 55) | 0.1 (0.08, 0.12) | |
| Eastern Europe | 7.68 (6.56, 8.98) | 32.17 (27.43, 37.7) | 7.33 (6.29, 8.5) | 31.89 (27.27, 37.2) | -0.01 (-0.05, 0.02) | |
| Central Europe | 4.28 (3.54, 5.23) | 32.93 (27.15, 40.14) | 4.23 (3.52, 5.12) | 32.76 (26.86, 39.87) | -0.02 (-0.03, -0.01) | |
| Central Asia | 1.45 (1.13, 1.82) | 22.52 (17.74, 28.07) | 2.06 (1.62, 2.59) | 22.22 (17.52, 27.74) | -0.07 (-0.08, -0.06) | |
| North Africa and Middle East | 16.08 (13.1, 19.73) | 49.5 (41.38, 59.22) | 31.88 (25.65, 39.25) | 51.36 (41.65, 62.67) | 0.2 (0.16, 0.23) | |
| South Asia | 28.55 (24.01, 33.99) | 30.23 (25.6, 35.58) | 54.67 (46.46, 64.4) | 30.46 (25.94, 35.47) | 0.23 (0.07, 0.39) | |
| Southeast Asia | 14.91 (12.33, 17.89) | 34.7 (28.9, 41.24) | 25.39 (21.03, 30.37) | 36.33 (30.24, 43.15) | 0.13 (0.1, 0.17) | |
| East Asia | 43.23 (36.38, 51.42) | 35.16 (29.89, 41) | 49.79 (42.82, 57.48) | 31.81 (27.12, 36.64) | -0.49 (-0.59, -0.39) | |
| Oceania | 0.23 (0.18, 0.29) | 39.07 (31.1, 48.44) | 0.5 (0.39, 0.63) | 40.07 (31.83, 49.9) | 0.08 (0.06, 0.09) | |
| Western Sub-Saharan Africa | 5.11 (4.11, 6.35) | 30.19 (24.88, 36.32) | 12.76 (10.26, 15.81) | 30.67 (25.33, 36.83) | 0.11 (0.06, 0.17) | |
| Eastern Sub-Saharan Africa | 5.76 (4.63, 7.14) | 36.94 (30.41, 44.94) | 13.39 (10.79, 16.68) | 37.16 (30.5, 45.31) | 0.01 (-0.02, 0.05) | |
| Central Sub-Saharan Africa | 1.8 (1.39, 2.28) | 38.65 (30.8, 48) | 4.48 (3.49, 5.67) | 38.64 (30.9, 48.26) | 0.01 (0, 0.01) | |
| Southern Sub-Saharan Africa | 1.75 (1.47, 2.11) | 36.42 (30.71, 42.77) | 2.86 (2.4, 3.4) | 36.58 (31, 43.08) | 0.02 (-0.02, 0.05) | |
| No., number; ASPR, age-standardized prevalence rate; UI, uncertainty interval; EAPC, estimated annual percentage change; CI, confidential interval. | | | | | | |

| **Table S2.** DALYs and age-standardized DALYs rate per 1000 people for anxiety disorders in 1990 and 2019, and its estimated annual percentage change from 1990 to 2019. | | | | | |
| --- | --- | --- | --- | --- | --- |
| **Variables** | **1990** | | **2019** | | **1990-2019** |
|  | **DALYs**  **No.×10^6^ (95% UI)** | **Age-standardized DALYs rate**  **per 1000**  **No. (95% UI)** | **DALYs**  **No.×10^6^ (95% UI)** | **Age-standardized DALYs rate**  **per 1000**  **No. (95% UI)** | **EAPC in age-standardized DALYs rate**  **No. (95% CI)** |
| **Overall** | 18.66 (12.9, 25.55) | 3.61 (2.51, 4.92) | 28.68 (19.86, 39.32) | 3.6 (2.49, 4.94) | -0.01 (-0.04, 0.02) |
| **Sex** |  |  |  |  |  |
| Males | 7.11 (4.92, 9.77) | 2.73 (1.9, 3.76) | 10.96 (7.63, 15.06) | 2.75 (1.91, 3.78) | 0.03 (-0.01, 0.08) |
| Females | 11.55 (7.99, 15.8) | 4.48 (3.11, 6.1) | 17.72 (12.24, 24.28) | 4.45 (3.07, 6.09) | -0.03 (-0.09, 0.03) |
| **SDI region** |  |  |  |  |  |
| High SDI | 3.83 (2.65, 5.27) | 4.41 (3.05, 6.08) | 4.91 (3.39, 6.71) | 4.57 (3.13, 6.27) | -0.02 (-0.15, 0.11) |
| High-middle SDI | 4.25 (2.96, 5.81) | 3.62 (2.52, 4.95) | 5.52 (3.84, 7.51) | 3.6 (2.5, 4.95) | 0.03 (0, 0.05) |
| Middle SDI | 5.93 (4.09, 8.17) | 3.56 (2.48, 4.84) | 9.1 (6.32, 12.42) | 3.63 (2.53, 4.98) | 0.05 (0.01, 0.08) |
| Low-middle SDI | 3.21 (2.19, 4.4) | 3.19 (2.22, 4.36) | 5.78 (3.99, 7.98) | 3.29 (2.29, 4.52) | 0.22 (0.17, 0.27) |
| Low SDI | 1.43 (0.97, 2) | 3.21 (2.19, 4.38) | 3.36 (2.25, 4.69) | 3.31 (2.27, 4.56) | 0.16 (0.12, 0.21) |
| **GBD region** |  |  |  |  |  |
| High-income Asia Pacific | 0.5 (0.35, 0.69) | 2.71 (1.88, 3.74) | 0.49 (0.34, 0.67) | 2.53 (1.74, 3.51) | -0.25 (-0.33, -0.17) |
| High-income North America | 1.46 (1.02, 1.98) | 4.86 (3.39, 6.59) | 2.03 (1.43, 2.75) | 5.22 (3.63, 7.09) | -0.09 (-0.4, 0.23) |
| Western Europe | 2.14 (1.46, 2.97) | 5.3 (3.61, 7.33) | 2.45 (1.66, 3.37) | 5.38 (3.65, 7.48) | 0.14 (0.09, 0.2) |
| Australasia | 0.12 (0.08, 0.17) | 5.69 (3.79, 7.94) | 0.17 (0.11, 0.24) | 5.76 (3.84, 8.06) | 0.13 (0.04, 0.22) |
| Tropical Latin America | 0.8 (0.56, 1.1) | 5.57 (3.91, 7.61) | 1.68 (1.17, 2.3) | 7 (4.87, 9.54) | 0.97 (0.54, 1.41) |
| Andean Latin America | 0.18 (0.12, 0.25) | 5.16 (3.45, 7.23) | 0.34 (0.22, 0.47) | 5.27 (3.51, 7.42) | 0.09 (0.07, 0.11) |
| Central Latin America | 0.51 (0.34, 0.71) | 3.36 (2.29, 4.62) | 0.97 (0.66, 1.34) | 3.76 (2.58, 5.21) | 0.37 (0.2, 0.53) |
| Southern Latin America | 0.24 (0.17, 0.34) | 4.94 (3.38, 6.83) | 0.34 (0.24, 0.46) | 4.91 (3.43, 6.62) | -0.03 (-0.06, 0) |
| Caribbean | 0.14 (0.09, 0.2) | 4.09 (2.72, 5.73) | 0.2 (0.14, 0.29) | 4.2 (2.79, 5.87) | 0.1 (0.08, 0.12) |
| Eastern Europe | 0.73 (0.51, 0.99) | 3.06 (2.15, 4.19) | 0.69 (0.49, 0.93) | 3.05 (2.15, 4.14) | 0.01 (-0.03, 0.04) |
| Central Europe | 0.41 (0.27, 0.57) | 3.14 (2.11, 4.39) | 0.4 (0.27, 0.55) | 3.13 (2.12, 4.35) | 0 (-0.01, 0) |
| Central Asia | 0.14 (0.09, 0.2) | 2.16 (1.45, 3.02) | 0.2 (0.13, 0.28) | 2.13 (1.43, 2.98) | -0.06 (-0.07, -0.06) |
| North Africa and Middle East | 1.56 (1.07, 2.15) | 4.74 (3.26, 6.52) | 3.07 (2.07, 4.31) | 4.92 (3.34, 6.85) | 0.2 (0.16, 0.23) |
| South Asia | 2.71 (1.88, 3.71) | 2.83 (1.98, 3.84) | 5.18 (3.63, 7.06) | 2.86 (2.01, 3.91) | 0.26 (0.09, 0.42) |
| Southeast Asia | 1.44 (0.98, 1.98) | 3.3 (2.29, 4.5) | 2.43 (1.68, 3.34) | 3.48 (2.42, 4.76) | 0.15 (0.12, 0.19) |
| East Asia | 4.2 (2.93, 5.78) | 3.38 (2.38, 4.64) | 4.76 (3.36, 6.51) | 3.08 (2.15, 4.22) | -0.47 (-0.57, -0.37) |
| Oceania | 0.02 (0.01, 0.03) | 3.71 (2.47, 5.21) | 0.05 (0.03, 0.07) | 3.8 (2.54, 5.32) | 0.08 (0.06, 0.1) |
| Western Sub-Saharan Africa | 0.49 (0.33, 0.68) | 2.88 (1.98, 4) | 1.24 (0.84, 1.72) | 2.93 (2.01, 4.06) | 0.13 (0.07, 0.19) |
| Eastern Sub-Saharan Africa | 0.55 (0.37, 0.77) | 3.49 (2.37, 4.79) | 1.29 (0.87, 1.82) | 3.54 (2.41, 4.88) | 0.05 (0.01, 0.08) |
| Central Sub-Saharan Africa | 0.17 (0.11, 0.24) | 3.63 (2.42, 5.1) | 0.43 (0.28, 0.62) | 3.66 (2.45, 5.17) | 0.04 (0.03, 0.05) |
| Southern Sub-Saharan Africa | 0.17 (0.12, 0.23) | 3.46 (2.41, 4.75) | 0.27 (0.19, 0.37) | 3.45 (2.41, 4.73) | 0 (-0.03, 0.03) |
| DALYs, Disability-Adjusted Life Years; No., number; UI, uncertainty interval; EAPC, estimated annual percentage change; CI, confidential interval. | | | | | |

| **Table S3.** The countries and territories with the ASIR/1000 in 2019 more than 8.0. | | | | |
| --- | --- | --- | --- | --- |
| **Number** | **Location** | **ASIR/1000 in 2019**  **No. (95% UI)** | **ASPR/1000 in 2019**  **No. (95% UI)** | **Age-standardized**  **DALYs rate /1000 in 2019**  **No. (95% UI)** |
| 1 | Iran | 10.27 (8.27, 12.44) | 72.68 (62.15, 85.02) | 6.96 (4.83, 9.55) |
| 2 | Portugal | 10.06 (7.73, 12.61) | 80.28 (63.90, 100.09) | 7.68 (5.04, 10.82) |
| 3 | Brazil | 9.94 (7.95, 12.14) | 74.10 (63.27, 86.39) | 7.03 (4.90, 9.59) |
| 4 | New Zealand | 9.75 (7.74, 11.93) | 72.63 (61.67, 86.16) | 6.92 (4.82, 9.41) |
| 5 | Norway | 9.45 (7.61, 11.35) | 67.36 (57.87, 78.43) | 6.44 (4.50, 8.84) |
| 6 | Ireland | 9.18 (7.17, 11.32) | 69.08 (55.44, 84.41) | 6.61 (4.38, 9.15) |
| 7 | Switzerland | 9.04 (7.04, 11.11) | 67.91 (55.14, 81.62) | 6.51 (4.38, 8.95) |
| 8 | Cyprus | 8.81 (6.87, 11.06) | 64.67 (51.26, 80.99) | 6.20 (4.13, 8.75) |
| 9 | Lebanon | 8.66 (6.76, 10.96) | 60.51 (48.11, 76.54) | 5.79 (3.86, 8.17) |
| 10 | Paraguay | 8.63 (6.67, 10.99) | 63.05 (50.78, 79.26) | 6.01 (3.99, 8.36) |
| 11 | Malta | 8.61 (6.73, 10.83) | 62.48 (49.99, 78.69) | 5.98 (3.97, 8.41) |
| 12 | France | 8.51 (6.60, 10.73) | 62.74 (49.91, 78.16) | 6.01 (4.00, 8.38) |
| 13 | Germany | 8.46 (6.61, 10.58) | 62.08 (49.84, 76.83) | 5.93 (3.90, 8.42) |
| 14 | Austria | 8.43 (6.67, 10.47) | 60.06 (48.15, 74.19) | 5.76 (3.87, 7.97) |
| 15 | Syria | 8.30 (6.44, 10.54) | 56.08 (44.51, 69.84) | 5.36 (3.59, 7.58) |
| 16 | Australia | 8.26 (6.37, 10.53) | 58.15 (46.40, 72.72) | 5.55 (3.67, 7.85) |
| 17 | Palestine | 8.23 (6.35, 10.40) | 55.46 (44.28, 69.52) | 5.29 (3.49, 7.42) |
| 18 | USA | 8.22 (6.57, 10.05) | 56.98 (48.43, 67.28) | 5.34 (3.73, 7.25) |
| 19 | Greece | 8.17 (6.32, 10.30) | 57.79 (46.57, 71.79) | 5.54 (3.71, 7.79) |
| 20 | Bolivia | 8.11 (6.27, 10.40) | 57.41 (46.19, 72.65) | 5.49 (3.63, 7.82) |
| 21 | Italy | 8.06 (6.49, 9.64) | 55.62 (47.55, 65.02) | 5.32 (3.73, 7.27) |
| 22 | Libya | 8.04 (6.32, 10.14) | 53.24 (42.77, 66.46) | 5.10 (3.41, 7.13) |
| ASIR, age-standardized incidence rate; ASPR, age-standardized prevalence rate; DALYs, disability-adjusted life years; No., number; UI, uncertainty interval. | | | | |

| **Table S4.** The countries and territories with the ASIR/1000 in 2019 less than 5.0. | | | | | |
| --- | --- | --- | --- | --- | --- |
| **Number** | **Location** | **ASIR/1000 in 2019**  **No. (95% UI)** | **ASPR/1000 in 2019**  **No. (95% UI)** | **Age-standardized**  **DALYs rate /1000 in 2019**  **No. (95% UI)** |  |
| 1 | Uzbekistan | 3.49 (2.73, 4.38) | 20.26 (15.94, 25.77) | 1.94 (1.30, 2.71) |  |
| 2 | Kyrgyzstan | 3.53 (2.76, 4.42) | 20.60 (16.37, 25.57) | 1.98 (1.34, 2.78) |  |
| 3 | Kazakhstan | 3.60 (2.80, 4.55) | 21.16 (16.40, 26.63) | 2.02 (1.34, 2.88) |  |
| 4 | Mongolia | 3.64 (2.85, 4.55) | 21.43 (17.05, 26.57) | 2.05 (1.38, 2.85) |  |
| 5 | Turkmenistan | 3.89 (3.04, 4.89) | 23.42 (18.74, 29.26) | 2.25 (1.50, 3.19) |  |
| 6 | Japan | 3.96 (3.26, 4.70) | 23.24 (19.87, 27.02) | 2.25 (1.58, 3.08) |  |
| 7 | Tajikistan | 3.98 (3.12, 5.07) | 24.04 (19.21, 29.94) | 2.31 (1.53, 3.21) |  |
| 8 | Vietnam | 4.03 (3.18, 5.05) | 22.87 (18.14, 28.27) | 2.20 (1.49, 3.13) |  |
| 9 | Azerbaijan | 4.06 (3.16, 5.10) | 24.78 (19.65, 30.65) | 2.38 (1.59, 3.33) |  |
| 10 | Georgia | 4.07 (3.14, 5.14) | 24.95 (19.97, 31.04) | 2.39 (1.60, 3.34) |  |
| 11 | Singapore | 4.53 (3.49, 5.68) | 27.08 (21.35, 33.70) | 2.63 (1.75, 3.72) |  |
| 12 | Mali | 4.59 (3.56, 5.78) | 27.26 (21.64, 33.97) | 2.61 (1.74, 3.69) |  |
| 13 | Mauritania | 4.61 (3.60, 5.83) | 27.46 (21.67, 34.28) | 2.64 (1.75, 3.73) |  |
| 14 | Brunei | 4.69 (3.60, 5.88) | 28.57 (22.62, 35.58) | 2.75 (1.86, 3.85) |  |
| 15 | Senegal | 4.79 (3.74, 6.09) | 28.93 (22.62, 35.89) | 2.77 (1.85, 3.90) |  |
| 16 | Armenia | 4.80 (3.72, 6.10) | 31.51 (25.23, 39.25) | 3.02 (2.01, 4.27) |  |
| 17 | India | 4.87 (3.96, 5.81) | 29.67 (25.53, 34.39) | 2.79 (1.96, 3.78) |  |
| 18 | Niger | 4.89 (3.81, 6.17) | 29.68 (23.32, 36.80) | 2.85 (1.87, 4.02) |  |
| 19 | Nigeria | 4.90 (3.98, 5.83) | 29.92 (25.69, 34.93) | 2.86 (2.01, 3.92) |  |
| 20 | Poland | 4.91 (3.98, 5.86) | 30.82 (26.38, 35.78) | 2.95 (2.08, 4.04) |  |
| 21 | Ghana | 4.91 (3.82, 6.17) | 30.01 (23.57, 37.35) | 2.88 (1.92, 4.12) |  |
| 22 | Czech | 4.92 (3.82, 6.19) | 31.54 (25.35, 39.09) | 3.01 (2.00, 4.21) |  |
| 23 | Ukraine | 4.99 (4.06, 5.99) | 31.33 (26.66, 36.41) | 3.00 (2.11, 4.14) |  |
| 24 | Russia | 4.99 (4.05, 5.97) | 31.34 (26.86, 36.42) | 2.99 (2.11, 4.08) |  |
| ASIR, age-standardized incidence rate; ASPR, age-standardized prevalence rate; DALYs, disability-adjusted life years; No., number; UI, uncertainty interval. | | | | | |

| **Table S5.** The countries and territories with the EAPC in ASIR from 1990 to 2019 more than 0.15. | | | | | |
| --- | --- | --- | --- | --- | --- |
| **Number** | **Location** | | **EAPC in ASIR**  **No. (95% CI)** | **EAPC in ASPR**  **No. (95% CI)** | **EAPC in age-standardized DALYs rate**  **No. (95% CI)** |
| 1 | Mexico | | 0.76 (0.56,0.96) | 0.89 (0.66,1.13) | 0.90 (0.66,1.15) |
| 2 | Brazil | | 0.53 (0.28,0.77) | 0.99 (0.54,1.44) | 1.00 (0.55,1.44) |
| 3 | Ireland | | 0.37 (0.24,0.51) | 0.48 (0.30,0.65) | 0.49 (0.30,0.67) |
| 4 | Spain | | 0.35 (0.22,0.48) | 0.71 (0.44,0.98) | 0.70 (0.43,0.97) |
| 5 | Turkey | | 0.34 (0.27,0.4) | 0.45 (0.36,0.54) | 0.45 (0.36,0.54) |
| 6 | Lebanon | | 0.34 (0.21,0.46) | 0.37 (0.21,0.54) | 0.40 (0.23,0.56) |
| 7 | Nepal | | 0.30 (0.27,0.34) | 0.39 (0.33,0.45) | 0.42 (0.36,0.47) |
| 8 | Chile | | 0.25 (0.13,0.37) | 0.10 (0.05,0.16) | 0.12 (0.06,0.18) |
| 9 | Bangladesh | | 0.22 (0.12,0.33) | 0.27 (0.15,0.39) | 0.29 (0.17,0.41) |
| 10 | Laos | | 0.22 (0.18,0.25) | 0.26 (0.21,0.31) | 0.29 (0.24,0.34) |
| 11 | Egypt | | 0.22 (0.04,0.41) | 0.25 (0.01,0.50) | 0.27 (0.02,0.51) |
| 12 | India | | 0.22 (0.02,0.43) | 0.23 (0.02,0.44) | 0.27 (0.05,0.48) |
| 13 | Kuwait | | 0.21 (0.16,0.26) | 0.29 (0.22,0.36) | 0.29 (0.22,0.35) |
| 14 | Australia | | 0.20 (0.10,0.29) | 0.2 (0.07,0.33) | 0.20 (0.07,0.33) |
| 15 | Syria | | 0.18 (0.14,0.21) | 0.22 (0.18,0.27) | 0.21 (0.17,0.24) |
| 16 | Uganda | | 0.16 (0.13,0.18) | 0.22 (0.19,0.26) | 0.28 (0.24,0.31) |
| 17 | Iraq | | 0.16 (0.10,0.22) | 0.2 (0.12,0.29) | 0.22 (0.15,0.30) |
| 18 | Israel | | 0.15 (-0.09,0.39) | 0.24 (-0.09,0.58) | 0.24 (-0.10,0.57) |
| 19 | Venezuela | | 0.15 (0.12,0.17) | 0.21 (0.17,0.24) | 0.21 (0.17,0.25) |
| 20 | Niger | | 0.15 (0.12,0.19) | 0.2 (0.16,0.24) | 0.22 (0.18,0.26) |
| ASIR, age-standardized incidence rate; ASPR, age-standardized prevalence rate; DALYs, disability-adjusted life years; No., number; EAPC, estimated annual percentage change; CI, confidential interval. | | | | | |
| **Table S6.** The countries and territories with the EAPC in ASIR from 1990 to 2019 less than -0.03. | | | | | |
| **Number** | **Location** | **EAPC in ASIR**  **No. (95% CI)** | | **EAPC in ASPR**  **No. (95% CI)** | **EAPC in Age-standardized DALYs rate**  **No. (95% CI)** |
| 1 | Japan | -0.5 (-0.59,-0.41) | | -0.58 (-0.67,-0.48) | -0.57 (-0.66,-0.47) |
| 2 | China | -0.34 (-0.42,-0.25) | | -0.51 (-0.61,-0.4) | -0.49 (-0.59,-0.38) |
| 3 | Colombia | -0.3 (-0.56,-0.04) | | -0.45 (-0.83,-0.06) | -0.44 (-0.82,-0.05) |
| 4 | Mongolia | -0.22 (-0.38,-0.05) | | -0.27 (-0.49,-0.06) | -0.26 (-0.48,-0.05) |
| 5 | Ethiopia | -0.17 (-0.23,-0.1) | | -0.24 (-0.33,-0.16) | -0.21 (-0.29,-0.12) |
| 6 | Taiwan, China | -0.16 (-0.19,-0.13) | | -0.16 (-0.2,-0.12) | -0.16 (-0.21,-0.12) |
| 7 | Vietnam | -0.11 (-0.3,0.09) | | -0.14 (-0.37,0.09) | -0.12 (-0.36,0.11) |
| 8 | Norway | -0.08 (-0.13,-0.04) | | -0.2 (-0.26,-0.13) | -0.18 (-0.25,-0.12) |
| 9 | Azerbaijan | -0.05 (-0.07,-0.02) | | -0.07 (-0.09,-0.04) | -0.06 (-0.09,-0.04) |
| 10 | Singapore | -0.05 (-0.09,-0.02) | | -0.11 (-0.14,-0.08) | -0.09 (-0.12,-0.06) |
| 11 | Somalia | -0.05 (-0.07,-0.04) | | -0.07 (-0.08,-0.05) | -0.05 (-0.06,-0.04) |
| 12 | New Zealand | -0.05 (-0.12,0.03) | | -0.07 (-0.14,0.01) | -0.06 (-0.14,0.02) |
| 13 | Georgia | -0.04 (-0.05,-0.02) | | -0.05 (-0.06,-0.04) | -0.06 (-0.08,-0.05) |
| 14 | Belarus | -0.04 (-0.04,-0.03) | | -0.05 (-0.06,-0.05) | -0.04 (-0.04,-0.04) |
| 15 | Maldives | -0.04 (-0.06,-0.02) | | -0.02 (-0.04,0.01) | 0.01 (-0.02,0.04) |
| 16 | Czech | -0.03 (-0.05,-0.02) | | -0.06 (-0.07,-0.05) | -0.07 (-0.08,-0.05) |
| 17 | Russia | -0.03 (-0.03,-0.02) | | -0.04 (-0.04,-0.04) | -0.02 (-0.02,-0.01) |
| 18 | Estonia | -0.03 (-0.04,-0.03) | | -0.05 (-0.06,-0.04) | -0.03 (-0.04,-0.02) |
| 19 | Slovakia | -0.03 (-0.04,-0.01) | | -0.03 (-0.05,-0.02) | -0.03 (-0.04,-0.01) |
| ASIR, age-standardized incidence rate; ASPR, age-standardized prevalence rate; DALYs, disability-adjusted life years; No., number; EAPC, estimated annual percentage change; CI, confidential interval. | | | | | |


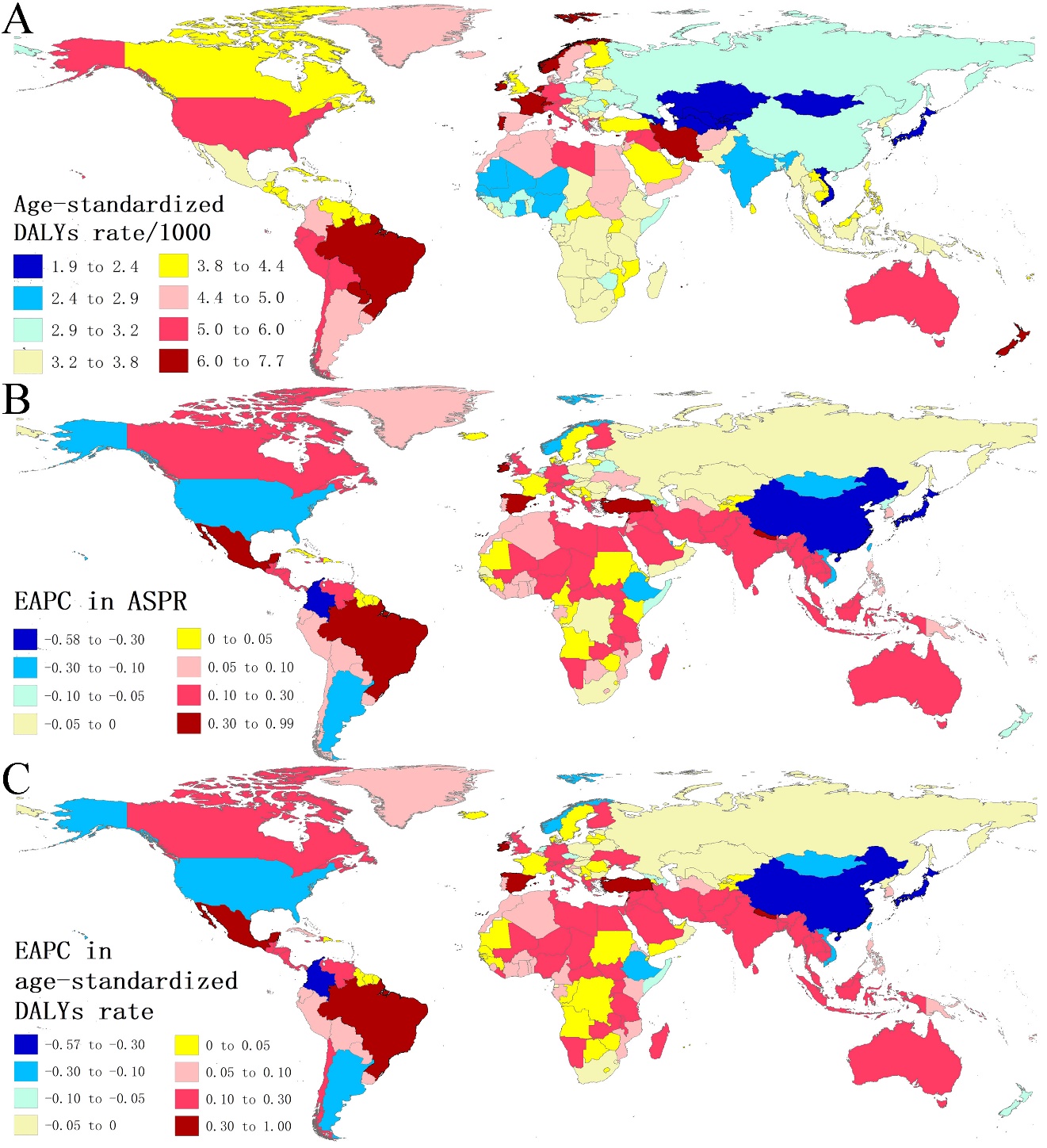


**Figure S1.** The global disease burden of anxiety disorders in 204 countries and territories. (A) The age-standardized DALYs rate in 2019; (B) The EAPC in ASPR from 1990 to 2019; (C) The EAPC in age-standardized DALYs rate from 1990 to 2019. ASIR, age-standardized incidence rate; DALYs, disability-adjusted life years; ASPR, age-standardized prevalence rate; EAPC, estimated annual percentage change.


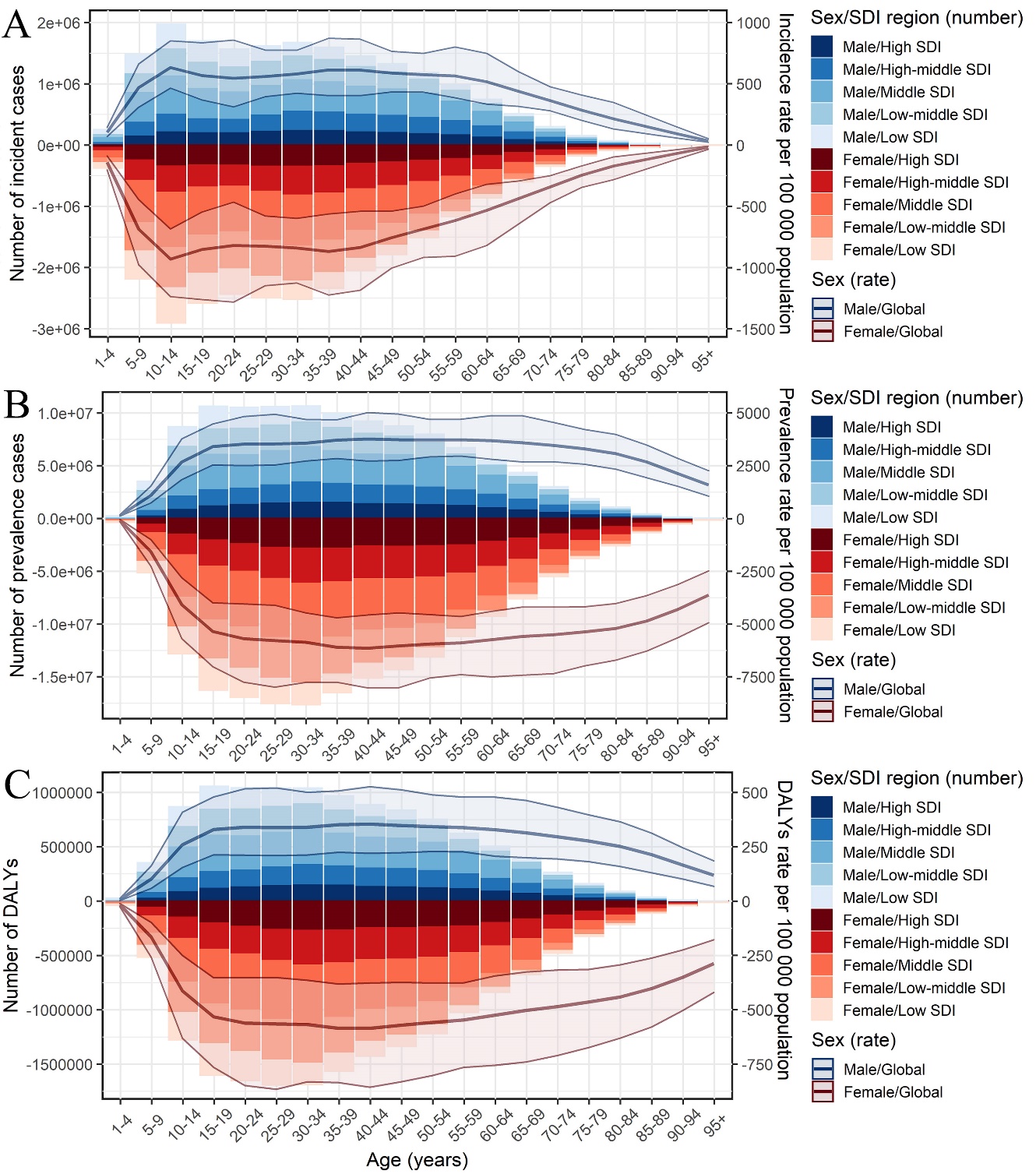


**Figure S2.** Age-specific counts and rates of anxiety disorders burden by sex and SDI regions, 2019. (A) incidence; (B) prevalence; (B) DALYs. SDI, Socio-demographic index; DALYs, disability-adjusted life years.


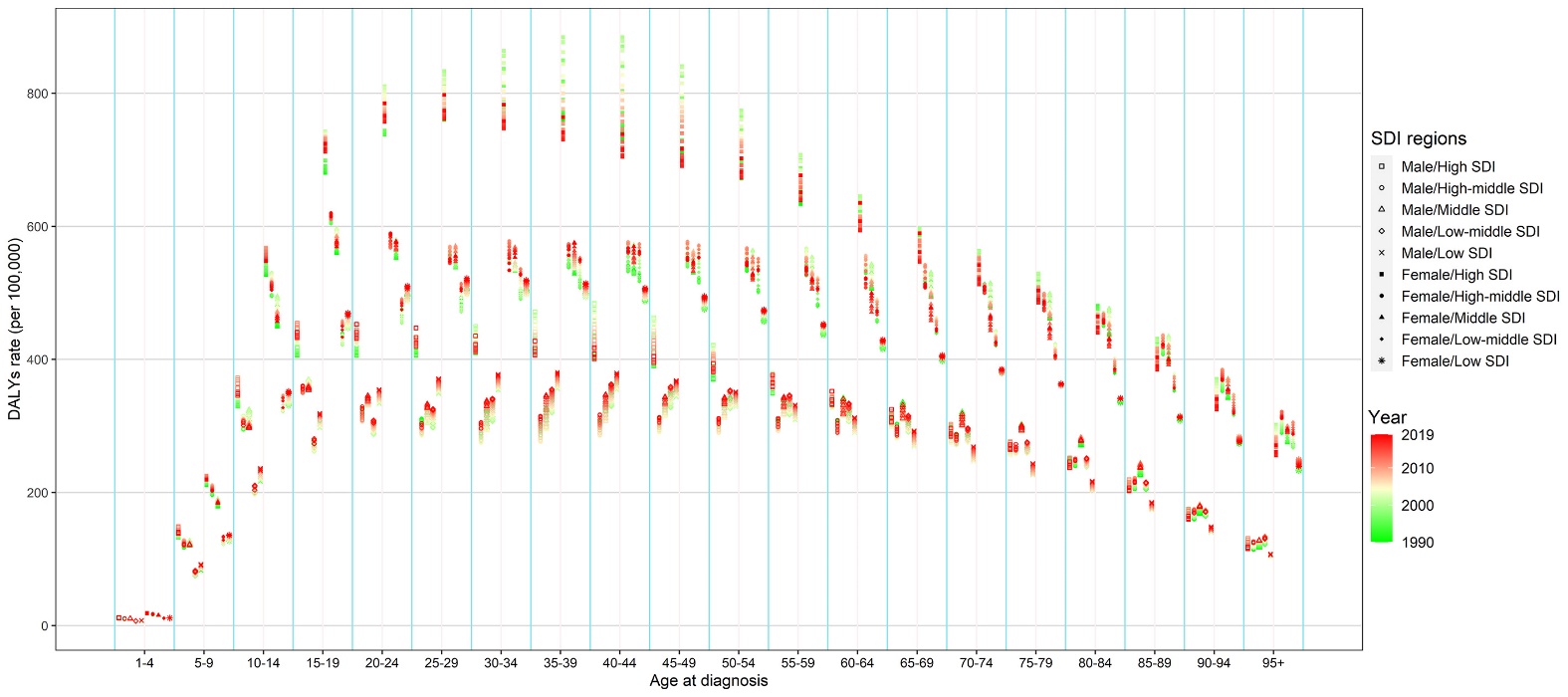
**Figure S3.** The annual DALYs rate of anxiety disorders by different age groups, two sexes, and SDI regions, from 1990 to 2019.


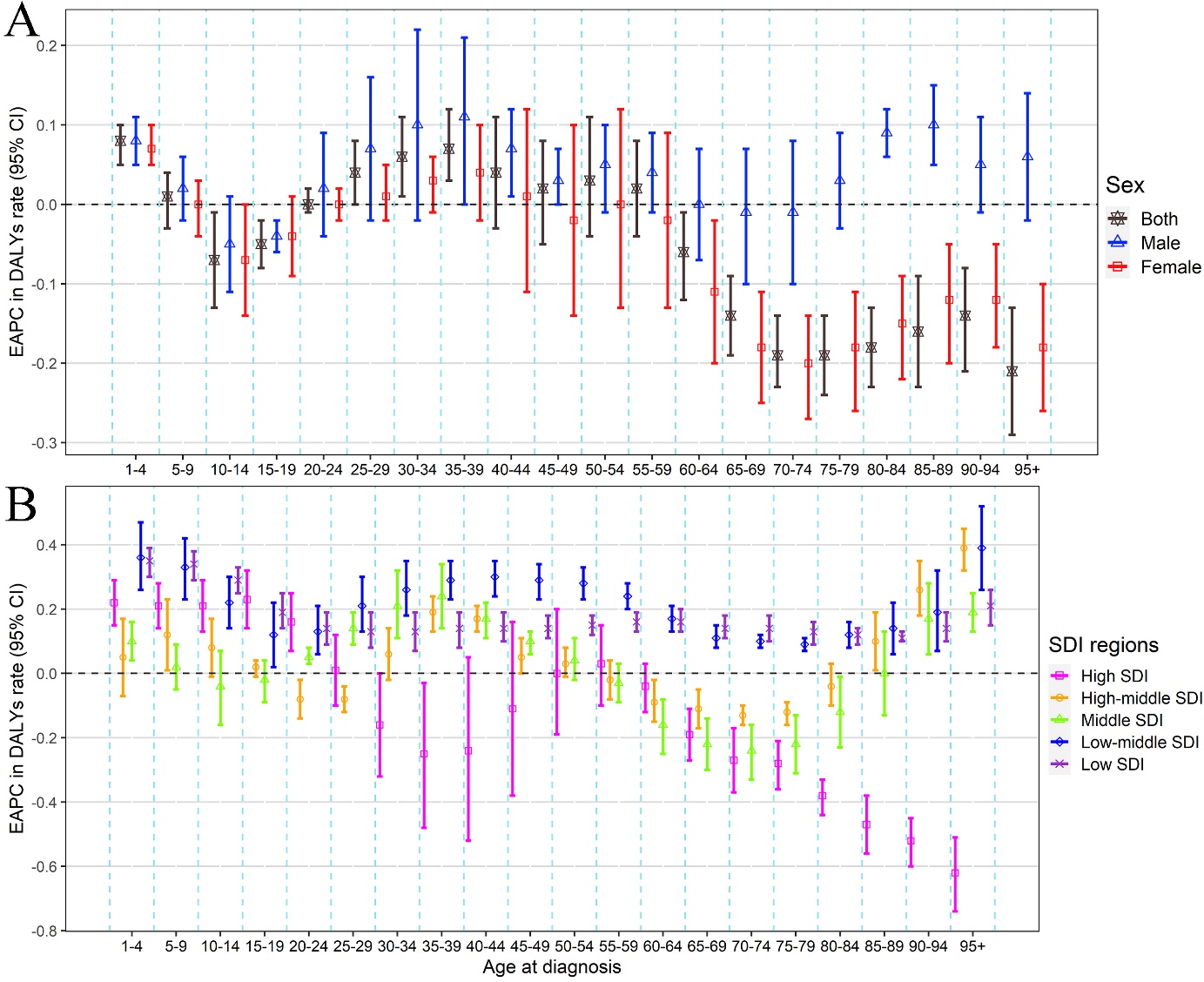


**Figure S4.** The change of the DALYs rate of anxiety disorders by different age groups, sexes, and SDI regions, from 1990 to 2019. (A) EAPC in DALYs rate by sexes; (B) EAPC in DALYs rate by SDI regions. DALYs, disability-adjusted life years; EAPC, estimated annual percentage change; SDI, Socio-demographic index.


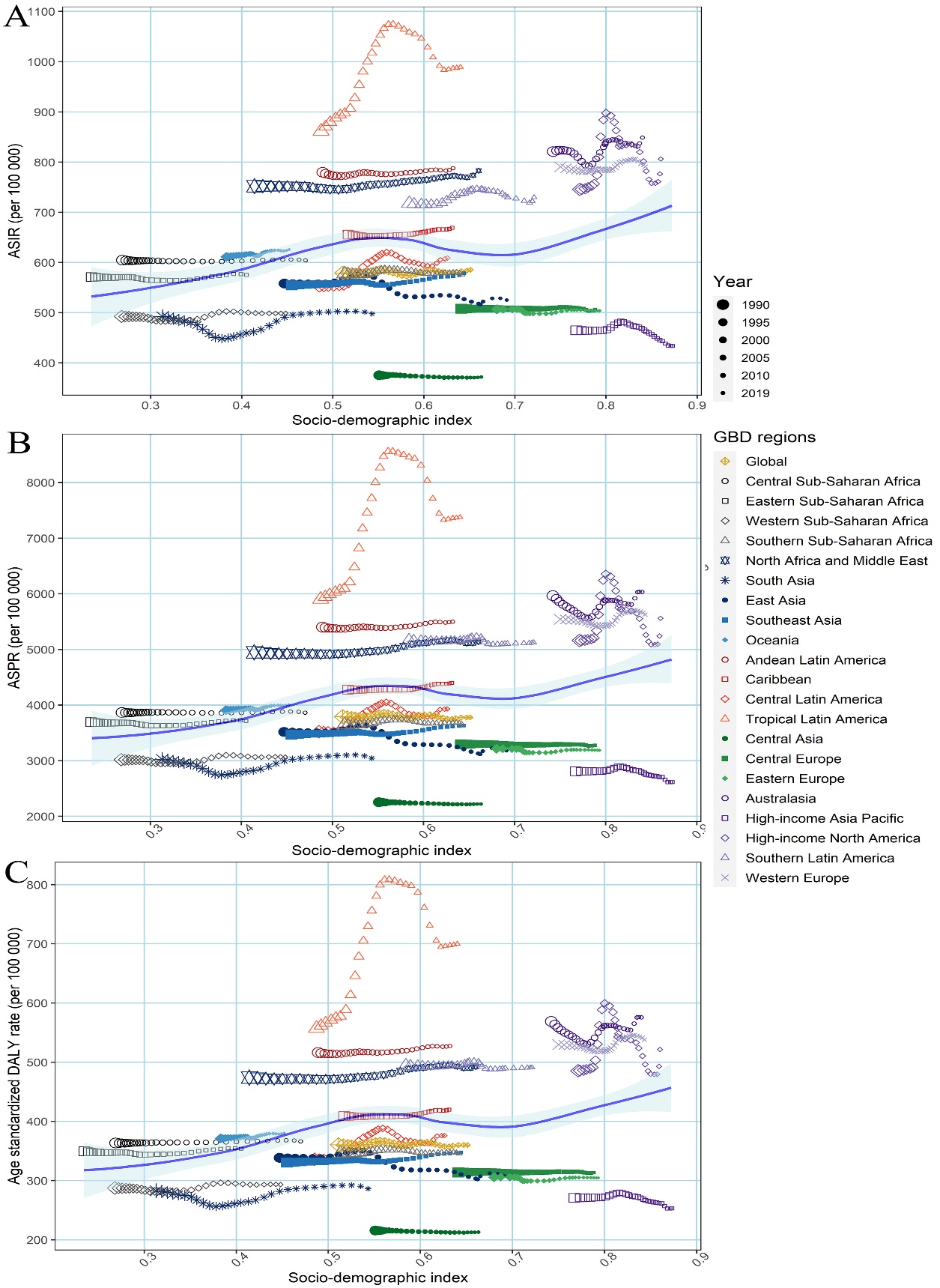


**Figure S5.** The annual burden in age-standardized burden rates of anxiety disorders across 21 GBD regions with SDI from 1990 to 2019. (A) ASIR; (B) ASPR; (C) age-standardized DALYs rate. GBD, global burden of disease; SDI, socio-demographic index; DALYs, disability-adjusted life years.
